# Phosphoglycerate dehydrogenase is required for kernel development and defines a predominant serine synthesis pathway in maize

**DOI:** 10.1093/plcell/koaf126

**Published:** 2025-05-22

**Authors:** Ying Zhang, Ruoxuan Li, Daocan Zheng, Jinhong Zhao, Ke Qing, Rongrong He, Zhaoxing Ma, Jie Chen, Nianguo Xue, Xing Tian, Enqi Wang, Jiameng Xu, Yubin Li, Bao-Cai Tan, Zhipeng Zhou, Chengyuan Wang, Jiaqiang Dong

**Affiliations:** The Key Laboratory of Plant Development and Environmental Adaptation Biology, Ministry of Education; Shandong Key Laboratory of Precision Molecular Crop Design and Breeding; State Key Laboratory of Microbial Technology; School of Life Sciences, Shandong University, Qingdao 266237, China; Shanghai Institute of Immunity and Infection, Chinese Academy of Sciences, Shanghai 201203, China; National Key Laboratory of Agricultural Microbiology, College of Life Science and Technology, Huazhong Agricultural University, Wuhan 430070, China; National Key Laboratory of Agricultural Microbiology, College of Life Science and Technology, Huazhong Agricultural University, Wuhan 430070, China; The Key Laboratory of Plant Development and Environmental Adaptation Biology, Ministry of Education; Shandong Key Laboratory of Precision Molecular Crop Design and Breeding; State Key Laboratory of Microbial Technology; School of Life Sciences, Shandong University, Qingdao 266237, China; National Key Laboratory of Agricultural Microbiology, College of Life Science and Technology, Huazhong Agricultural University, Wuhan 430070, China; The Key Laboratory of Plant Development and Environmental Adaptation Biology, Ministry of Education; Shandong Key Laboratory of Precision Molecular Crop Design and Breeding; State Key Laboratory of Microbial Technology; School of Life Sciences, Shandong University, Qingdao 266237, China; The Key Laboratory of Plant Development and Environmental Adaptation Biology, Ministry of Education; Shandong Key Laboratory of Precision Molecular Crop Design and Breeding; State Key Laboratory of Microbial Technology; School of Life Sciences, Shandong University, Qingdao 266237, China; The Key Laboratory of Plant Development and Environmental Adaptation Biology, Ministry of Education; Shandong Key Laboratory of Precision Molecular Crop Design and Breeding; State Key Laboratory of Microbial Technology; School of Life Sciences, Shandong University, Qingdao 266237, China; The Key Laboratory of Plant Development and Environmental Adaptation Biology, Ministry of Education; Shandong Key Laboratory of Precision Molecular Crop Design and Breeding; State Key Laboratory of Microbial Technology; School of Life Sciences, Shandong University, Qingdao 266237, China; College of Agronomy, Qingdao Agricultural University, Qingdao 266109, China; The Key Laboratory of Plant Development and Environmental Adaptation Biology, Ministry of Education; Shandong Key Laboratory of Precision Molecular Crop Design and Breeding; State Key Laboratory of Microbial Technology; School of Life Sciences, Shandong University, Qingdao 266237, China; College of Agronomy, Qingdao Agricultural University, Qingdao 266109, China; The Key Laboratory of Plant Development and Environmental Adaptation Biology, Ministry of Education; Shandong Key Laboratory of Precision Molecular Crop Design and Breeding; State Key Laboratory of Microbial Technology; School of Life Sciences, Shandong University, Qingdao 266237, China; National Key Laboratory of Agricultural Microbiology, College of Life Science and Technology, Huazhong Agricultural University, Wuhan 430070, China; Shanghai Institute of Immunity and Infection, Chinese Academy of Sciences, Shanghai 201203, China; The Key Laboratory of Plant Development and Environmental Adaptation Biology, Ministry of Education; Shandong Key Laboratory of Precision Molecular Crop Design and Breeding; State Key Laboratory of Microbial Technology; School of Life Sciences, Shandong University, Qingdao 266237, China

## Abstract

Serine functions as both a substrate for protein biosynthesis and a signaling molecule for growth and development. However, the mechanism remains poorly understood. Here, we cloned and functionally characterized the maize (*Zea mays*) gene *Dek20*, which encodes phosphoglycerate dehydrogenase1 (PGDH1), the rate-limiting enzyme in the phosphorylated pathway of serine biosynthesis (PPSB). The *dek20*(Ser282Leu) mutation disrupts the interaction between residues Ser282 and His284, leading to the release of His284, which subsequently binds NAD^+^/NADH to inhibit serine biosynthesis. Consequently, serine content decreases dramatically, and the cellular response to nutrient starvation is enriched in transcriptome analysis. Serine deficiency triggers tRNA^Ser^ degradation and reduced translation elongation at serine codons. The stalled ribosomes activate General Control Nonderepressible 2 (GCN2) kinase, which affects the phosphorylation of eukaryotic initiation factor 2α (eIF2α) and ribosomal protein S6 kinase (S6 K), furtherly inhibiting translation initiation. Consistent with these findings, polysome profiling and Ribo-seq analysis revealed a marked decrease in translation efficiency in *dek20*. Notably, proteins essential for storage compound biosynthesis and cell cycle progression exhibit reduced translation in *dek20*. Collectively, our findings reveal the primary serine biosynthesis pathway and a mechanism for monitoring amino acid levels in maize, the model plant with C4 photosynthesis.

## Teaser

Phosphoglycerate dehydrogenase defines the primary serine biosynthesis pathway in maize, a model C4 photosynthetic plant, which differs from the pathway in C3 plants.

## Introduction

L-Serine (serine) is a crucial amino acid that serves as an essential component of proteins. Additionally, serine is involved in the synthesis of several biomolecules necessary for cell division and growth, including nucleotides, phospholipids, sphingolipids, and other amino acids (Ros et al. 2014). Serine also functions as a single-carbon donor in tetrahydrofolate metabolism, providing methyl groups (Kalhan and Hanson 2012). Moreover, serine is the precursor of D-serine, which acts as an endogenous signaling molecule in mammalian brain communication (Mothet et al. 2000) and facilitates communication between pollen and pistil tissues in plants (Michard et al. 2011). Therefore, maintaining appropriate serine levels in nearly all tissues is critical for proper growth and development. (Ser or serine is short for L-serine below)

In bacteria and mammals, serine is primarily synthesized via the phosphorylated pathway of serine biosynthesis (PPSB), which begins with the glycolytic intermediate 3-phosphoglycerate (3-PGA) (Pizer 1963; Snell 1984; Dey et al. 2005). PPSB consists of 3 catalytic steps (Amelio et al. 2014; Ros et al. 2014). The first step, catalyzed by 3-phosphoglycerate dehydrogenase (PGDH), oxidizes 3-PGA into 3-phosphohydroxypyruvate (3-PHP) and is the rate-limiting reaction. Next, 3-PHP is converted to 3-phosphoserine (3-PS) by 3-phosphoserine aminotransferase (PSAT) via transamination, with glutamate serving as the amino group donor. Finally, 3-PS is converted into serine by 3-phosphoserine phosphatase (PSP). PGDH, the rate-limiting enzyme, is a research hotspot in oncology due to its high expression in various cancers, including rat hepatomas and human colon carcinoma (Snell 1984; Snell and Weber 1986; Snell et al. 1988). In 2011, it was discovered that the *PGDH* gene is duplicated and upregulated, diverting metabolic flux from glycolysis to serine metabolism to promote cancer progression (Locasale et al. 2011; Possemato et al. 2011). Since then, research has focused on the regulation of serine synthesis, particularly the enzymatic activity of PGDH, as a target for cancer therapy (Chaneton et al. 2012; Kalhan and Hanson 2012; Sun et al. 2015; Mullarky et al. 2016; Pacold et al. 2016; Yang and Vousden 2016; Tajan et al. 2021).

In contrast, serine is long believed to be generated mainly through the glycolate pathway in plants, which takes place in mitochondria and is associated with photorespiration (Douce et al. 2001; Bauwe et al. 2010; Maurino and Peterhansel 2010). The serine content is decreased dramatically when grown in elevated concentrations of CO_2_, which inhibits photorespiration and blocks the glycolate pathway (Zimmermann et al. 2021; Rosa-Téllez et al. 2024). In addition to the glycolate pathway, plants possess all the genes essential for the PPSB pathway. The *Arabidopsis thaliana* genome has 3 genes encoding PGDH (At4g34200, *PGDH1*; At1g17745, *PGDH2*; At3g19480, *PGDH3*), 2 for PSAT (At4g35630, *PSAT1*; At2g17630, *PSAT2*), and 1 for PSP (At1g18640, *PSP1*) (Ros et al. 2014). Though the *pgdh1* and *psp1* mutants exhibit embryo lethality and defective male gametophyte development, the serine content is not decreased in these mutants (Benstein et al. 2013; Cascales-Miñana et al. 2013; Toujani et al. 2013).

In comparison, maize (*Zea mays*), a model plant with C4 photosynthesis, exhibits low rates of photorespiration (Sage et al. 2012; Wang et al. 2014). As a result, the well-established photorespiration-related glycolate pathway may play a limited role in maize. In accordance, serine content is highest in the immature sections of maize leaves, where the photosynthetic apparatus is not yet fully developed (Wang et al. 2014). Additionally, the developing maize kernel, a non-photosynthetic organ comprising the embryo and endosperm, shows active cell division and growth, both of which demand substantial amounts of serine. Therefore, the precise mechanism of serine biosynthesis in maize remains unclear.

Amino acids have traditionally been viewed primarily as building blocks for protein synthesis. However, recent studies have increasingly highlighted the major role of amino acid metabolism in organogenesis and morphogenesis (Guo et al. 2021; Kawade et al. 2023). Additionally, amino acid metabolism is crucial for the biosynthesis of storage proteins (Holding et al. 2010; Gaufichon et al. 2017; Lee et al. 2020; Huang et al. 2022). Research in mammals and yeast shows that amino acid deficiency triggers a response that increases the availability of uncharged tRNAs, which activate General Control Nonderepressible 2 (GCN2) kinase, leading to the phosphorylation of eukaryotic initiation factor 2α (eIF2α) and mammalian target of rapamycin (mTOR) (Dever et al. 1992; Shu et al. 2020). Phosphorylation of eIF2α inhibits the recycling of inactive eIF2-GDP to active eIF2-GTP for the assembly of the initiator ternary complex, while mTOR phosphorylation decreases its kinase activity, suppressing ribosomal protein S6 kinase (S6K) phosphorylation, and ultimately inhibiting translation initiation. However, the precise mechanism of this response in plants remains unclear.

In this study, we investigated the classical *defective kernel* (*dek*) mutant, *dek20*, which was initially generated through ethyl methanesulfonate (EMS) mutagenesis (Neuffer and Sheridan 1980). By employing bulked segregant analysis in combination with exome sequencing (Dong et al. 2019), we identified *Dek20* as encoding phosphoglycerate dehydrogenase, the rate-limiting enzyme in the PPSB pathway. Metabolomic analysis revealed that the *dek20* mutation led to a significant reduction in serine content, indicating that PPSB is the primary serine synthesis pathway in maize, the model plant with C4 photosynthesis. Structural analysis of DEK20 showed that the residue Ser at 282 is important for its catalytic activity. Serine deficiency in *dek20* led to degradation of tRNA^Ser^, and reduced translation elongation at serine codons. The stalled ribosomes activated GCN2 to repress the translation of proteins essential for storage compound synthesis and cell cycle progression. This suggests the existence of a monitoring system for amino acid content in plants. Collectively, these findings indicate that DEK20 is essential for maize kernel development by participating in serine synthesis.

## Results

### *Dek20* mutation leads to embryo lethality and reduced endosperm

**Figure 1.**
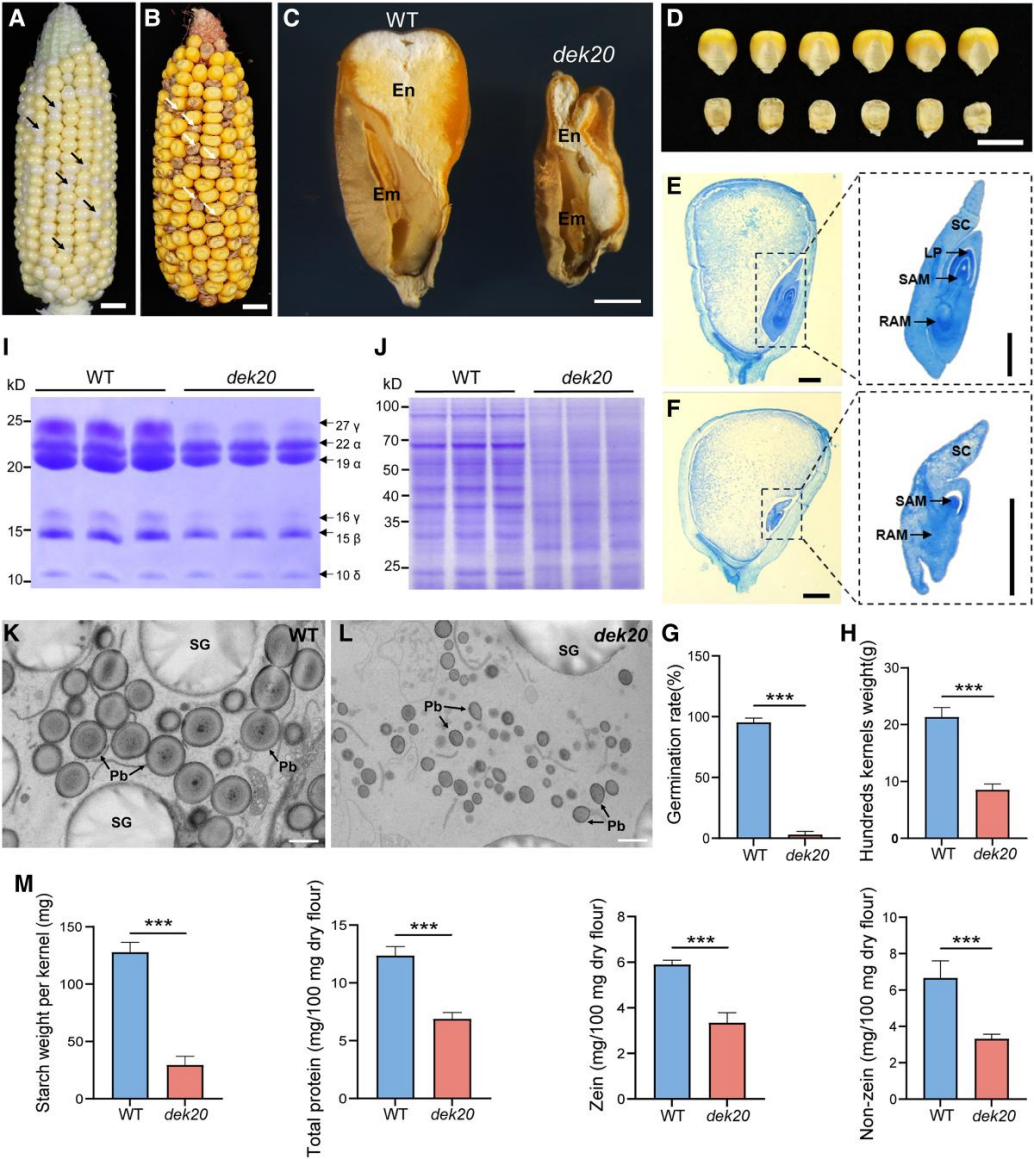
Phenotypic analysis of maize *dek20* mutant. **A** and **B)** Self-pollinated 14 DAP (A) and mature (B) ears from BC_3_F_1_ heterozygous plant. Arrows indicate *dek20* kernels. Scale bar, 1 cm. **C)** Longitudinal sections of WT and *dek20* mature kernels. En, endosperm; Em, embryo. WT, wild type. Scale bar, 1 mm. **D)** WT and *dek20* kernels on the germinal side. Scale bar, 1 cm. **E** and **F)** Longitudinal paraffin sections of WT (E) and *dek20* (F) kernels at 14 DAP. Embryos are enlarged to show the details (Images were digitally extracted for comparison). SC, scutellum; LP, leaf primordia; SAM, shoot apical meristem; RAM, root apical meristem. Scale bar, 0.5 mm. **G)** Germination test of WT and *dek20* mature kernels. Values are means ± SD (*n* = 3, kernels from 3 independent ears; ***P < 0.001 as determined by two-tailed *t-*test). **H)** Comparison of the 100-kernel weight of randomly selected mature WT and *dek20* kernels in a segregated population. Values are means ± SD (*n* = 3, kernels from 3 independent ears; ***P < 0.001 as determined by two-tailed *t-*test). **I)** SDS-PAGE Analysis of zein proteins in WT and *dek20* kernels. The size of zein protein is indicated next to it. **J)** SDS-PAGE Analysis of non-zein proteins in WT and *dek20* kernels. **K** and **L)** Transmission electron microscopy observations of starchy endosperm cells of WT (K) and *dek20* (L) kernels at 18 DAP. PB, protein body; SG, starch granule. Scale bar, 1 *μ*m. **M)** Comparison of contents of starch, total protein, zein and non-zein protein in WT and *dek20* kernels. Values are means ± SD (*n* = 3, kernels from 3 independent ears; ***P < 0.001 as determined by two-tailed *t-*test).

The *dek20* (*dek20*-*N1392A*) was obtained from the Maize Genetics Cooperation Stock Center and previously generated through EMS mutagenesis (Neuffer and Sheridan 1980). This mutant was backcrossed to the B73 inbred line 3 times and subsequently self-pollinated to obtain the BC_3_F_2_ ears. The segregation ratio of kernels with wild-type (WT) (+/+ and *dek20*/+) or mutant (*dek20*/*dek20*) phenotype approached 3:1, suggesting that *dek20* contained a recessive mutation in a single gene (Supplementary Fig. S1). The mutant phenotype became distinctive at 14 days after pollination (DAP) (Fig. 1A), with developing mutant kernels appearing white and transparent. Upon maturity, *dek20* kernels exhibited a small and flattened morphology, with both the endosperm and the embryo severely impaired (Figs. 1B to 1D). To further investigate the developmental defects of *dek20*, paraffin sections of both WT and mutant kernels, harvested from the same ear at 10 DAP, 14 DAP, and 18 DAP were performed (Figs. 1, E and F, Supplementary Fig. S2). Histological analysis revealed that the *dek20* embryo and endosperm exhibited a significant delay in differentiation compared to WT. Consistent with this finding, germination assays indicated that the majority of *dek20* kernels failed to germinate, highlighting the profound impact of the *Dek20* mutation on kernel development (Fig. 1G). Furthermore, the 100-kernel weight of *dek20* was 8.54 g, only 40% of that of WT (Fig. 1H). A significant reduction of starch, zein, and non-zein proteins was also observed in *dek20* (Figs. 1, I and J, and 1 m). Transmission electron microscopy (TEM) revealed that the *dek20* endosperm contained fewer and smaller starch granules and protein bodies compared to WT (Figs. 1 K and 1L). Collectively, these results indicate that *Dek20* is essential for kernel development.

### Cloning of the *Dek20* gene

**Figure 2.**
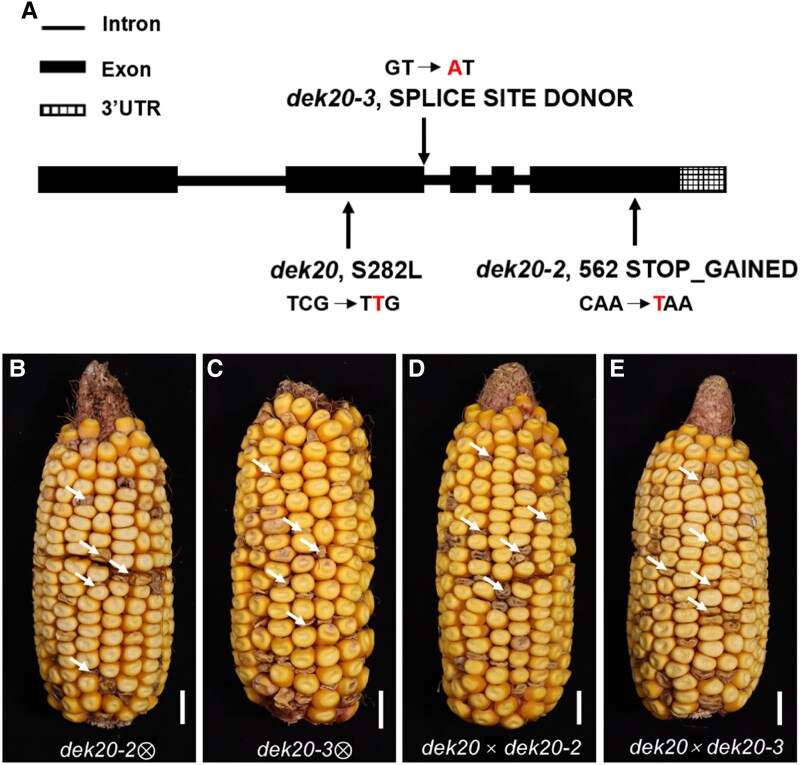
Identification and characterization of *Dek20* gene. **A)** Gene structure and mutation site of the *Dek20* gene. Lines represent introns, black boxes represent exons, and white boxes with grids represent 3′ untranslated regions. **B** and **C)** Self-pollinated mature ears from heterozygous *dek20-2* (B) and *dek20-3* (C) plants. Arrows indicate *dek20-2* or *dek20-3* kernels. Scale bars, 1 cm. **D** and **E)** Allelism tests between heterozygous *dek20* (*dek20*/+) and heterozygous *dek20-2* (D) or *dek20-3* (E) plants. Arrows indicate *dek20/dek20-2* or *dek20/dek20-3* kernels. Scale bar, 1 cm.

To identify the causal gene underlying the *dek20* mutation, genomic DNA pools were separately collected from the developing 14 DAP kernels of WT and mutant phenotype from the same ear. Exome sequencing was conducted to uncover variants, which were then filtered against the third-generation *Zea mays* haplotype map (Bukowski et al. 2018; Dong et al. 2019). Among the candidate mutations listed in Supplementary Data Set 1, a C-to-T mutation was identified in the second exon of *Zm00001d002051*, resulting in a substitution of the 282nd amino acid, from serine (Ser) to leucine (Leu), in *dek20* (Fig. 2A).

Next, to validate this causal mutation, 2 additional allelic mutants of *Zm00001d002051* were obtained from the Maize EMS-induced Mutant Database, *dek20*-*2* (*EMS3-02265d*) and *dek20*-*3* (*EMS3-1701bc*) (Lu et al. 2018). The *dek20-2* allele contained a C-to-T substitution in the fifth exon, leading to a premature stop codon, while *dek20-3* had a G-to-A substitution at the splice donor site of the second intron, resulting in mis-splicing that introduced a stop codon (Fig. 2A). All kernels carrying these homozygous mutated alleles exhibited similar defective kernel phenotypes (Figs. 2, B and C). Allelism tests were subsequently performed by crossing these alleles. The observed 3:1 segregation pattern in F_1_ ears confirmed the allelic relationship between *dek20* and *dek20-2* or *dek20-3* (Figs. 2, D and E, Supplementary Data Set 2). Collectively, our results demonstrate that *Zm00001d002051* is the *Dek20* gene.

### *Dek20* encodes a phosphoglycerate dehydrogenase

The genomic DNA sequence of *Dek20* is 2,877 bp in length and comprises 5 exons and 4 introns (Fig. 2A). The mature transcript of *Dek20* features an 1,881-bp coding sequence, which encodes a protein consisting of 626 amino acids that includes 3 domains: the substrate binding domain (SBD, residues 1-179), the nucleotide-binding domain (NBD, residues 180-367), and the regulatory domain (RBD, residues 388-626) (Supplementary Fig. S3A). Homology analysis indicated that DEK20 is the homolog of phosphoglycerate dehydrogenase (PGDH), which acts as a rate-limiting enzyme in the PPSB of bacteria and mammals. A phylogenetic tree was then constructed using the full-length protein sequences of DEK20 along with potential homologous proteins from diverse organisms. Notably, the DEK20 protein exhibited a high degree of sequence similarity to homologs in *Oryza* and *Arabidopsis*, PGDH1 (Supplementary Figs. 3B and 4).

### *Dek20* is primarily expressed in developing kernels

**Figure 3.**
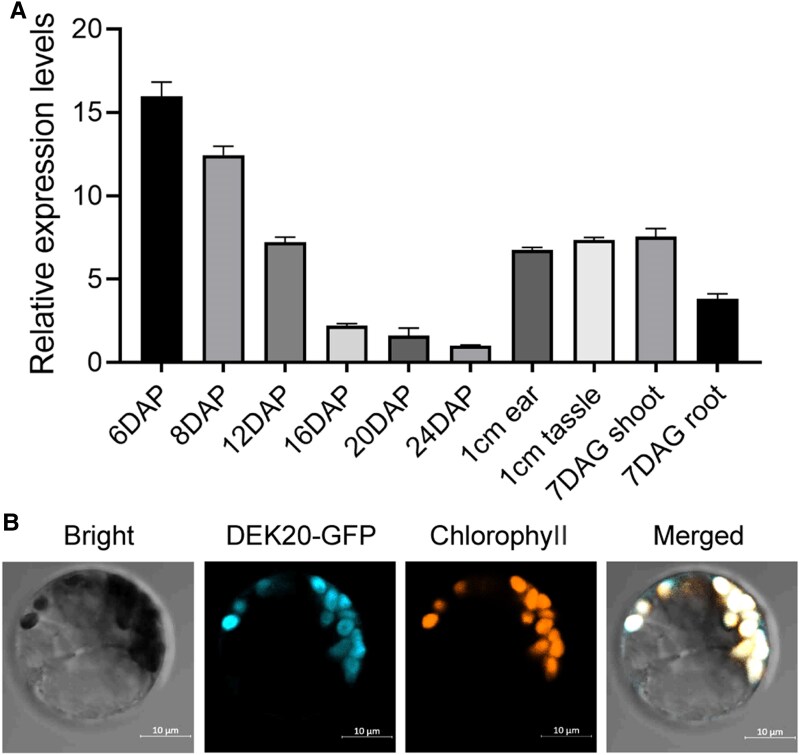
Expression pattern and subcellular localization of *Dek20*. **A)** RT-qPCR analysis of relative transcript levels of *Dek20* in maize. DAP means days after pollination. The whole kernel with embryo and endosperm was used. DAG means days after germination. Values are means ± SD (*n* = 3, kernels from 3 independent ears). **B)** Subcellular localization analysis of DEK20 protein in maize protoplast. Chlorophyll autofluorescence was used as a marker for chloroplastic localization.

To elucidate the expression patterns of the *Dek20* gene, we analyzed the existing transcriptome data (Chen et al. 2014; Zhan et al. 2015; Yi et al. 2019; Fu et al. 2023) and then performed RT-qPCR to verify. *Dek20* was expressed in a wide range of maize tissues, including root, shoot, shoot apical meristem, developing ear, and tassel, with particularly high expression levels in kernels (Fig. 3A, and Supplementary Fig. S5A). In the kernel, *Dek20* expression is most prominent in the starchy endosperm, aleurone layer, and embryo.

In maize, 4 genes encoded PGDHs with different expression patterns (Supplemental Figs. 3B, and 5B). *Dek20* (*PGDH1*) was primarily expressed in developing kernels, while *PGDH3* was preferentially expressed in developing tassels, ears, and seedlings. The expression level of *PGDH2* was very low, and *PGDH4* was undetectable. The expression of the other 3 *PGDH* genes remains unchanged in *dek20*, indicating that they cannot compensate for the role of DEK20 (Supplementary Fig. S5C). Subcellular localization analysis revealed that DEK20 proteins were located in plastids within maize protoplasts (Fig. 3B).

### DEK20 is required for serine biosynthesis

**Figure 4.**
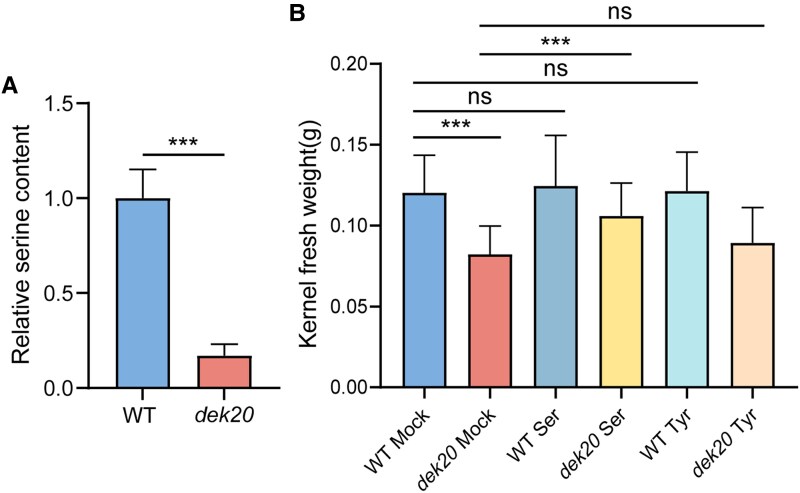
Serine content is decreased in *dek20* kernels. **A)** Relative free serine content of 14 DAP kernels in *dek20* compared with WT. Values are means ± SD (*n* = 3, kernels from 3 independent ears; ***P < 0.001 as determined by two-tailed *t*-test). **B)** Kernel fresh weight analysis after cultivated in medium supplemented with 3 mm L-serine or 3 mm L-Tyrosine. Values are means ± SD (*n* = 30, biologically independent kernels; ***P < 0.001; ns, no significant difference as determined by two-tailed *t*-test).

Since *Dek20* encodes PGDH, a homolog of the rate-limiting enzyme in the serine synthesis pathway of bacteria and mammals, to identify the effect of the *dek20* mutation, we employed ultra-performance liquid chromatography coupled with mass spectrometry (UPLC-MS) to analyze the contents of primary metabolites in 14 DAP kernels. In the metabolome analysis, a total of 507 primary metabolites were detected, of which 82 were differentially enriched (variable importance in projection (VIP) ≥ 1, foldchange ≥ 2 or foldchange ≤ 0.5, Supplementary Data Set 3). Among these, 52 metabolites were upregulated and 30 were downregulated. These differential metabolites were categorized into 6 groups based on their chemical properties (Supplementary Data Set 3). Among the amino acids and their derivatives, serine is the only amino acid that showed a significant decrease, with its content reduced by 80% (Fig. 4A). In contrast, 20 amino acid derivatives, such as L-Ornithine, were upregulated in *dek20*. Additionally, 23 lipids, 9 nucleotides and their derivatives, 14 organic acids, 6 vitamins, and 8 saccharides were differentially enriched. This aligns with serine's role in the biosynthesis of key biomolecules required for cell proliferation, including other amino acids, nitrogenous bases, phospholipids, and sphingolipids (Ros et al. 2014).

Moreover, since PPSB is a key branch of the glycolytic pathway, it may influence both the glycolytic and TCA pathways by diverting the flux of 3-PGA. To explore this, we first analyzed the contents of metabolites in both the glycolytic and TCA pathways, though they did not pass the statistical threshold (Supplementary Data Set 3). Unexpectedly, we found that most metabolites in the glycolytic pathway were decreased, including 3-PGA, which is a direct precursor of serine biosynthesis. However, in the TCA pathway, most metabolites remained unchanged or were upregulated in *dek20*. It has been reported that PPSB functions not only in serine biosynthesis but also in the provision of α-ketoglutaric acid, via the anaplerotic reactions that drive glutamine-derived carbon into the TCA cycle to counterbalance biosynthetic efflux in human (Possemato et al. 2011). However, the content of α-ketoglutaric acid was unaffected in 14 DAP *dek20* kernels, highlighting a key difference between plant and human metabolism.

To further verify whether the *dek20* phenotype is caused by serine deficiency, *dek20* kernels at 4 DAP were cultured on a medium supplemented with 3 mm serine. After 12 days of cultivation, the weight of individual *dek20* kernels significantly increased with serine supplementation compared to *dek20* kernels without supplementation or those supplemented with tyrosine (Fig. 4B). This result suggests that externally supplied serine can be metabolized, at least to some extent, in a manner similar to endogenously synthesized serine.

### The S282L mutation impairs the biochemical function of DEK20 protein

To elucidate the molecular mechanism underlying enzymic activity, we first purified the full-length DEK20 for crystallization. However, no crystal was obtained due to relatively flexible interactions between RBD and SBD. Consequently, we purified a truncated version (residues 1-387, referred to as DEK20^WT^), which includes only the SBD and the NBD. This approach successfully yielded crystals, which were solved in the apo form at a resolution of 3.5 Å (PDB ID: 9JCM). The statistics of data collection and model refinement are summarized in Supplementary Data Set 4.

**Figure 5.**
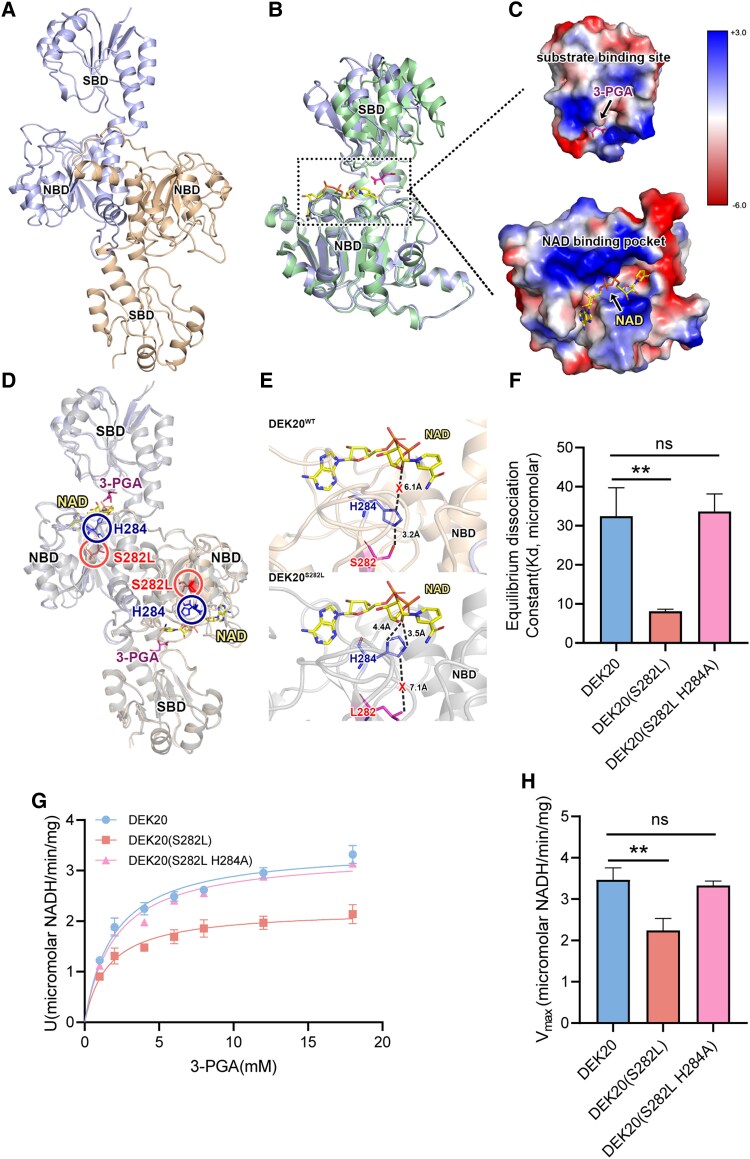
The DEK20(S282L) mutation affects the enzymic activity of DEK20 protein. **A)** Overview of crystal structure of DEK20^WT^ homodimer. Each monomer is colored differently. **B)** Structural superposition of human PGDH (green, PDB ID 2G76) and DEK20^WT^ (cyan). **C)** The electrostatic potential surface of cofactor and substrate binding sites. The upper insert shows the map for 3-PGA binding site and the lower insert shows the NAD^+^ binding pocket. The color bar represents the electrostatic potential ranging from −6 to +3 kT/e, with negative potentials shown in red and positive potentials in blue. **D)** Illustration of DEK20^S282L^ showing its Leu282 and His284 position. Leu282 and His284 are highlighted in red and blue, respectively. **E)** Close-up views of the superposition of NAD^+^ to DEK20^WT^ and DEK20^S282L^. Upper: Ser282 forms a hydrogen bond with His284, keeping imidazole group of histidine away from NAD^+^. Lower: Leu282 has no interaction with His284 resulting in the spatial position of the His284 imidazole group close to NAD^+^. Interacting residues are labeled and shown as sticks. Black dashed lines indicate H-bonds. The red X indicates no interaction. **F)** Equilibrium dissociation constants of different DEK20 proteins. Values are means ± SD (*n* = 3, biologically independent experiments; **P < 0.01; ns, no significant difference as determined by two-tailed *t*-test). **G)** Enzymic analysis of different DEK20 proteins. For each sample, three independent biological experiments were performed. Values are means ± SD. **H)**  *V*_max_ of different DEK20 proteins. Values are means ± SD (*n* = 3, biologically independent experiments; **P < 0.01; ns, no significant difference as determined by two-tailed *t*-test).

DEK20^WT^ forms a homodimer, with each monomer composed of 1 NBD and 1 SBD (Fig. 5A). Both domains exhibit conserved architectures: the SBD contains 5 α helices and 5 β strands, while the NBD consists of 7 α helices surrounding a central β sheet. Superimpositions of the structure with human 3-phosphoglycerate dehydrogenase (PDB ID 2G76) yielded a root-mean-square deviation (R.M.S.D.) of 1.42 Å, indicating similar overall conformations (Fig. 5B). Previous studies have shown that the PGDH family (EC 1.1.1.95) shares a conserved substrate binding mode. Indeed, both the superimposed cofactor (NAD^+^/NADH) and the substrate (3-PGA) from human 3-phosphoglycerate dehydrogenase fit well within our structure. The 3-PGA is situated in a cleft between NBD and SBD, stabilized by a series of positive residues such as arginine and histidine, while NAD^+^ occupies the binding pocket formed by the NBD (Figs. 5, B and C).

To elucidate the mechanism of reduced catalytic activity of DEK20(S282L) mutation, we purified and crystallized the same truncated fragment but with S282L mutation (residues 1-387, referring as DEK20^S282L^, PDB ID: 9JCN) (Fig. 5D). The overall structure of DEK20^S282L^ was quite similar to DEK20^WT^ (R.M.S.D. 0.465 Å). According to the structures, the residue Ser or Leu at 282 was located in NBD but had no direct interaction with NAD ^+^ . Interestingly, a conserved residue His at 284 among different species was located between NAD^+^ and Ser282. A more detailed comparison showed 2 distinguished interactions between NAD^+^ and these 2 residues. In the structure of DEK20^WT^, Ser282 formed a hydrogen bond with His284 (3.2 Å) keeping the imidazole group of histidine away from NAD ^+^ . By contrast, Leu282 had no interaction with His284 in DEK20^S282L^, resulting in the spatial position of the His284 imidazole group close to NAD^+^ (Fig. 5E). This change enabled His284 to interact with NAD^+^ through hydrogen bonds, which may increase the binding affinity and affect the release of NAD^+^ from DEK20(S282L), and eventually decrease the catalytic activity.

To verify whether the hypothesis is true, we assessed the binding affinity of DEK20 and DEK20(S282L) to its cofactor NAD^+^. We employed isothermal titration calorimetry with a microscale thermophoresis assay (MST) (Supplementary Fig. S6). This analysis quantitatively evaluated the interaction between DEK20 and NAD^+^. The dissociation constant (Kd) of DEK20 was found to be 32.45 *μ*M, while for DEK20(S282L), it is 8.11 *μ*M (Fig. 5F). These results indicate that the S282L mutation significantly enhances DEK20's binding ability to NAD^+^. To determine whether this enhancement resulted from the interaction of His284 with NAD^+^, we mutated His284 to Ala (H284A) and observed that the H284A mutation reversed the increase of NAD^+^ affinity of DEK20(S282L) (Fig. 5F).

To check whether the enzymic function of DEK20 is affected by the mutation, the recombinant DEK20 and DEK20(S282L) proteins were incubated in an in vitro reaction system. In this enzymic assay, NADH was generated through the oxidation of 3-PGA to 3-PHP. PGDH activity was quantitatively determined by monitoring the increase in NADH absorbance at 340 nm. Enzyme activity was standardized as the amount required to reduce 1 *μ*mol of NAD^+^ per minute under the specified conditions. Notably, the wild-type DEK20 exhibited superior enzyme activity compared to its mutated variant, DEK20(S282L) (Figs. 5, G and H).

Correspondingly, the decreased enzyme activity of DEK20(S282L) was also restored by the H284A mutation (Figs. 5, G and H). These findings demonstrate that Ser282 is critical for enzyme activity, facilitating its interaction with His284 to keep His284 away from the cofactor, NAD^+^. Both Ser282 and His284 are conserved across multiple organisms, providing valuable insights into the enzymic function and regulation of PGDH.

### Serine deficiency affects the stability of tRNA^Ser^ in *dek20*

**Figure 6.**
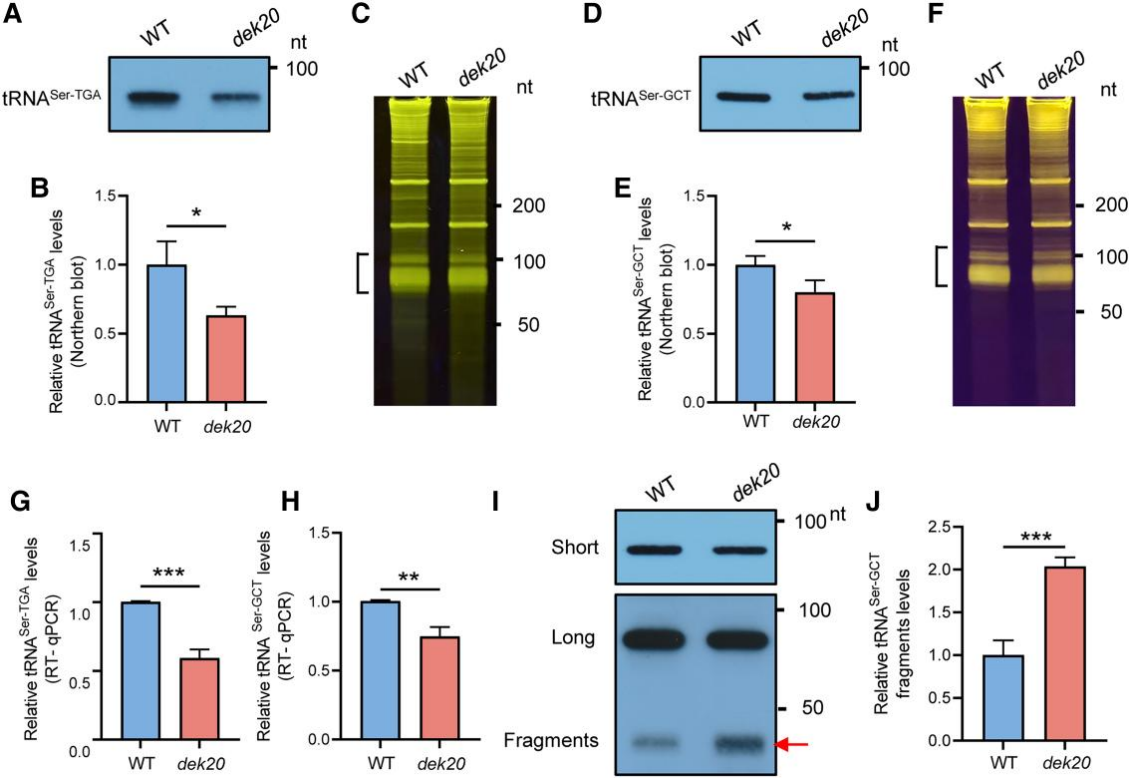
tRNA^Ser^ is decreased in *dek20* kernels. **A** and **B)** Northern blot results showing tRNA^Ser-TGA^ levels in WT and *dek20* kernels at 14 DAP. Values are means ± SD (*n* = 3, kernels from 3 independent ears; *P < 0.05 as determined by two-tailed *t*-test). **C)** Entire TBE-urea gel stained with SYBR gold, serving as loading control for Fig. 6A. **D** and **E)** Northern blot results showing tRNA^Ser-GCT^ levels in WT and *dek20* kernels at 14 DAP. Values are means ± SD (*n* = 3, kernels from 3 independent ears; *P < 0.05 as determined by two-tailed *t*-test). **F)** Entire TBE-urea gel stained with SYBR gold, serving as loading control for Fig. 6D. **G** and **H)** The relative levels of total tRNA^Ser-TGA^ and tRNA^Ser-GCT^ were measured by RT-qPCR in WT and *dek20* kernels at 14 DAP. Values are means ± SD (*n* = 3, kernels from 3 independent ears; *P < 0.05 as determined by two-tailed *t*-test). **I** and **J)** Plots showing tRNA fragments from tRNA^Ser-GCT^. Northern blots were exposed longer time to detect fragments. The arrow indicates tRNA^Ser-GCT^ fragments. Short, short exposure; long, long exposure. Values are means ± SD (*n* = 3, kernels from 3 independent ears, ***P < 0.001 as determined by two-tailed *t*-test).

During protein synthesis, amino acids are first charged to tRNAs by aminoacyl-tRNA synthetases before being delivered to ribosomes. tRNAs are destabilized following amino acid starvation or various stresses in eukaryotes (Lee and Collins 2005; Jöchl et al. 2008; Thompson et al. 2008; Megel et al. 2019). Interestingly, both tRNA^Ser-TGA^ and tRNA^Ser-GCT^ levels were found to be lower in *dek20* kernels (Fig. 6A to F). With RT-qPCR, we indeed found that the levels of tRNA^Ser-TGA^ and tRNA^Ser-GCT^ are decreased in *dek20* (Fig. 6, G and H). This decrease may result from the rapid degradation of serine tRNAs, leading to a reduced steady-state level. In accordance, we found that the degraded fragments of tRNA^Ser-GCT^ are much more accumulated in *dek20* than WT (Figs. 6, I and J).

### Serine deficiency represses protein translation in *dek20*

**Figure 7.**
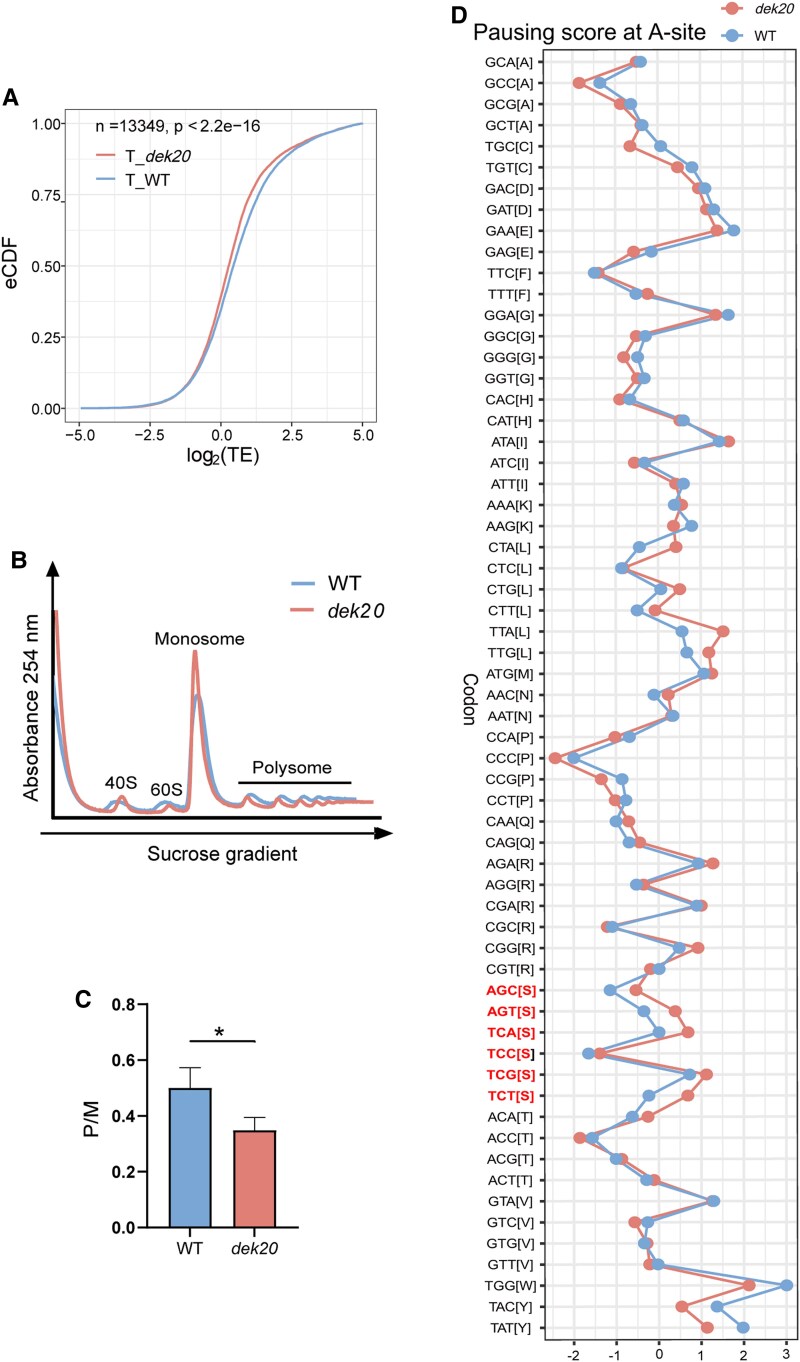
Protein translation is generally inhibited in *dek20*. **A)** Empirical cumulative distribution function (eCDF) of translation efficiency. **B)** Polysome profiling of 14 DAP kernels of WT and *dek20*. Peaks are labeled corresponding to 40S small subunit (40S), 60S large subunit (60S), monosome, and polysome. **C)** Plot of polysome/monosome ratios. P, polysome; M, monosome. Values are means ± SD (*n* = 3, kernels from 3 independent ears; *P < 0.05 as determined by two-tailed *t*-test). **D)** Plot of pausing scores for individual codon at the ribosomal A-site in *dek20* versus WT kernels.

To investigate the effect of the *dek20* mutation on protein translation, we conducted transcriptome and translatome analyses with 14 DAP WT and *dek20* kernels. The *dek20* mutation had a significant impact on both the transcriptome and translatome. At the transcriptional level, 2,265 genes were upregulated, while 2,267 genes were downregulated (|Foldchange|≥1.5, *p*_adj_ < 0.05, Supplementary Fig. S7, Supplementary Data Set 5). At the translational level, 1,548 genes were upregulated, and 1,535 genes were downregulated (|Fold change,| ≥ 1.5 and *p*_adj_ < 0.05, Supplementary Figs. S8 and S9, Supplementary Data Set 6). Translational efficiency (TE) is a key metric for assessing how effectively mRNAs are translated into proteins (Ingolia et al. 2009; Dunn et al. 2013). Our results indicated that TE was generally decreased in *dek20* (Fig. 7A). To confirm the translational repression, polysome profiling was performed with 14 DAP kernels. The ratio of polysomes to monosomes was analyzed to quantify the mRNA fraction engaged in active translation. The ratio was found to be significantly decreased in *dek20* compared to WT (Figs. 7, B and C), clearly demonstrating genome-wide repression of translation in *dek20*.

To understand how serine deficiency affects translation, we utilized Ribo-seq data to calculate pausing scores for all 61 amino acid-coding codons, a widely used metric for translation elongation speed. Consistent with the decreased serine levels in *dek20*, the pausing scores at ribosomal A-site for serine codons were higher than those in WT, while the pausing scores for other codons were either decreased or unchanged (Fig. 7D). However, the *dek20* mutation had little to no effect on the pausing scores of serine codons at P- or E-site (Supplementary Fig. S10). Collectively, these data indicate that the decreased levels of serine, together with the degradation of tRNA^Ser^, in *dek20* leads to reduced translation elongation at serine codons.

**Figure 8.**
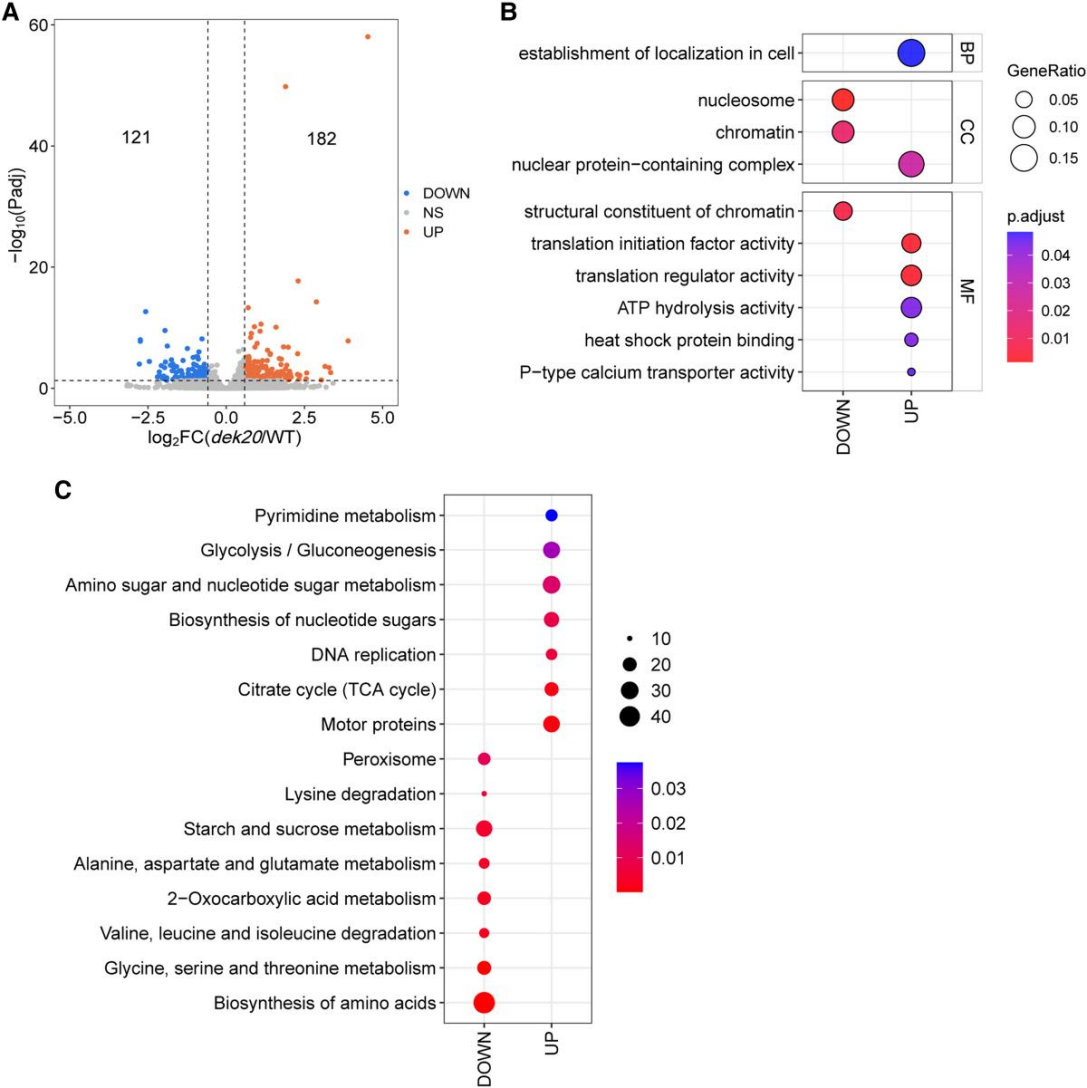
Analysis of differential translation efficiency genes in *dek20*. **A)** Volcano plot of differential translation efficiency genes (DTEGs) in *dek20* versus WT kernels. Differential translation efficiency defined by |Fold change| ≥ 1.5 and padj < 0.05. **B)** GO enrichment analysis of DTEGs in *dek20* versus WT kernels. Color scale indicates *P* value thresholds; dot size indicates gene ratio for each GO term, which helps assess the relative significance of each GO term, with higher values indicating a stronger correlation of that term in the enrichment analysis. **C)** KEGG enrichment analysis of DTEGs in *dek20* versus WT kernels. Color scale indicates *P* value thresholds; dot size indicates gene number for each pathway.

To investigate the impact of translation inhibition, we first identified genes that met a stringent threshold for TE differences between WT and *dek20*, as shown in Fig. 8A and Supplementary Data Set 7. Functional Gene Ontology (GO) enrichment analysis revealed that genes with increased TE were associated with biological processes such as cellular localization, cellular components like nuclear protein-containing complexes, and molecular functions including translation regulator activity and heat shock protein binding (Fig. 8B, Supplementary Data Set 8). Genes with decreased TE were enriched in cellular components like the nucleosome and molecular functions related to chromatin structural components (Fig. 8B, Supplementary Data Set 8). Kyoto Encyclopedia of Genes and Genomes (KEGG) pathway analysis revealed that genes with increased TE were associated with motor proteins, glycolysis and citrate cycle, amino sugar, and nucleotide sugar metabolism, while genes with decreased TE were significantly enriched in amino acid metabolic processes, particularly those related to glycine, serine, and threonine metabolism (Fig. 8C).

**Figure 9.**
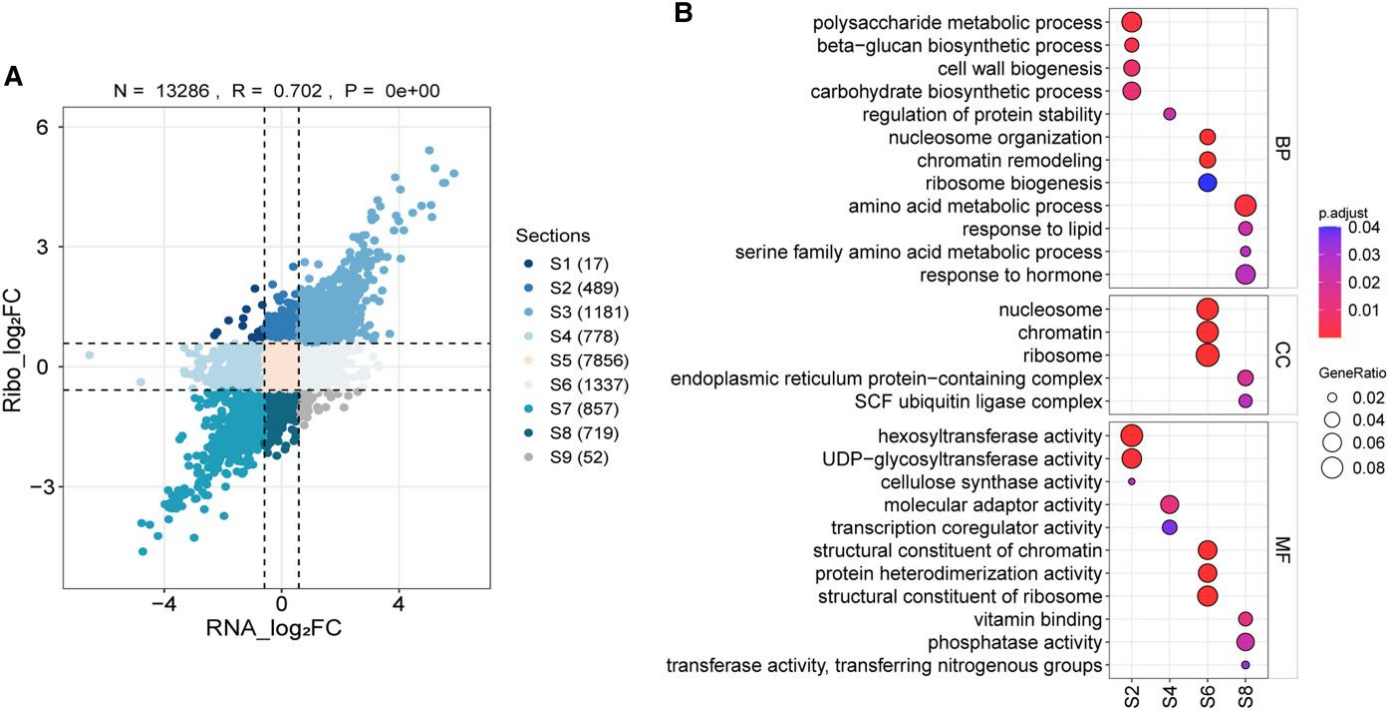
The comparison analysis between transcriptome and translatome. **A)** The nine-quadrant diagram. Nine squares in different colors indicated nine responsive groups (|Foldchange| ≥ 1.5). Classification of genes based on fold changes of RPFs and mRNAs. S1: Transcriptionally downregulated and translationally upregulated genes; S2: Transcriptionally unchanged and translationally upregulated genes; S3: Transcriptionally and translationally upregulated genes; S4: Transcriptionally downregulated and translationally unchanged genes; S5: Transcriptionally and translationally unchanged genes; S6: Transcriptionally upregulated and translational unchanged genes; S7: Transcriptionally and translationally downregulated genes; S8: Transcriptionally unchanged and translationally downregulated genes; S9: Transcriptionally upregulated and translationally downregulated genes. **B)** GO enrichment analysis of genes showing discordant regulation. Color scale indicates *p*adj value thresholds; dot size indicates gene ratio for each pathway, which helps assess the relative significance of each GO term, with higher values indicating a stronger correlation of that term in the enrichment analysis.

Since the number of genes passing this stringent threshold was relatively small and may lack statistical power, we adopted a less stringent approach. We compared RNA transcript levels with ribosome footprint levels to identify genes showing discordant regulation, and potential indicators of translational enhancement or repression in *dek20* (Fig. 9A, Supplementary Data Set 9). Further analysis of these groups revealed distinct GO enrichment patterns (Fig. 9B, Supplementary Data Sets 7). Class S1 comprised 17 genes that were transcriptionally downregulated but translationally upregulated, while Class S9 included 52 genes with the opposite pattern. Classes S2 and S8 represented genes regulated at the translational level without corresponding changes at the mRNA level. Specifically, Class S2 (489 translationally upregulated genes) was enriched in biological processes such as polysaccharide biosynthesis, and molecular functions like hexosyltransferase activity. In contrast, Class S8 (719 translationally downregulated genes) was enriched in biological processes such as amino acid metabolic processes, response to hormones, and cellular components like endoplasmic reticulum protein-containing complexes. Classes S4 and S6 reflected transcriptional changes without significant translational regulation. Class S4 (778 transcriptionally downregulated genes) was enriched in biological processes such as regulation of protein stability, the molecular function of protein adaptor and transcription coregulator activities, intracellular transport, and nutrient response. Class S6 (1,337 transcriptionally upregulated genes) was enriched in biological processes such as ribosome biogenesis, chromatin remodeling, molecular function like structural constituents of chromosomes and ribosomes, and cellular components like the chromosome and ribosome.

Together, both analyses consistently indicate that translationally downregulated genes are enriched in amino acid metabolic processes, particularly those involving glycine, serine, and threonine metabolism (Supplementary Data Set 10).

### Serine deficiency changed the phosphorylation status of eukaryotic initiation factor 2α and ribosomal protein S6 kinase in *dek20*

**Figure 10.**
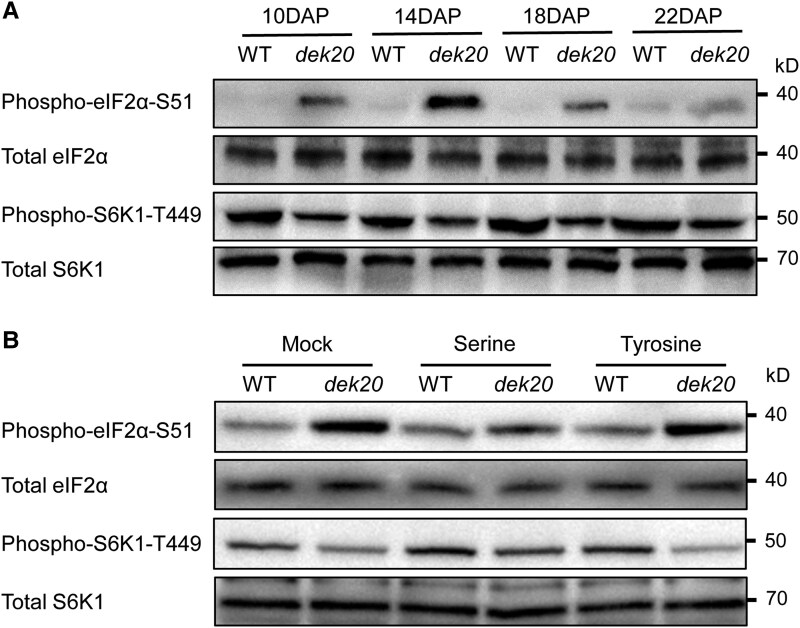
eIF2α and S6K1 phosphorylation analysis. **A)** Immunoblot analysis of eIF2α and S6K1 phosphorylation in WT and *dek20* kernel. **B)** Immunoblot analysis of eIF2α and S6K1 phosphorylation in WT and *dek20* kernels after cultivated in medium supplemented with 3 mm L-serine or 3 mm L-Tyrosine.

Amino acid depletion activates GCN2 kinase, leading to the phosphorylation of eIF2α and suppression of translation initiation in yeast and mammals (Kilberg et al. 2005; Shu et al. 2020). In Arabidopsis, similar responses have been observed: upon amino acid starvation or other stress conditions, the Arabidopsis GCN2 kinase (AtGCN2) is activated and phosphorylates eIF2α, resulting in global inhibition of protein synthesis (Lageix et al. 2008; Zhang et al. 2008; Li et al. 2013; Luna et al. 2014; Lokdarshi and von Arnim 2022). In addition to the GCN2-eIF2α pathway, amino acid depletion also impacts the mTOR signaling pathway in yeast and mammals, leading to reduced phosphorylation of S6 kinase (S6K)(Hay and Sonenberg 2004; Yuan et al. 2017; Shu et al. 2020). In plants, phosphorylation of S6K at threonine 449 (T449) is similarly required for its activation and downstream function, and this modification is dependent on endogenous target of rapamycin kinase activity (Xiong and Sheen 2012; Xiong et al. 2013). Additionally, reduced translation elongation also activates GCN2 directly by recruiting it to stalled ribosomes (Misra et al. 2024), which may feedback to repress translation initiation by phosphorylating eIF2α. Consistent with the decreased serine and tRNA^Ser^ levels, and also the reduced translation elongation at serine codons, we found a significant increase in eIF2α phosphorylation, and a decrease in S6 K phosphorylation in *dek20* at 10, 14, 18, and 22 DAP (Fig. 10A, Supplementary Fig. S11). Furthermore, following kernel cultivation in a medium supplied with 3 mm serine, the phosphorylation level of eIF2α decreased, while that of S6K increased (Fig. 10B, Supplementary Fig. S12). Collectively, these results suggest that serine deficiency alters the phosphorylation status of eIF2α and S6K, which may contribute to the decreased protein content observed in *dek20*.

### Serine deficiency affects storage compound formation in *dek20*

**Figure 11.**
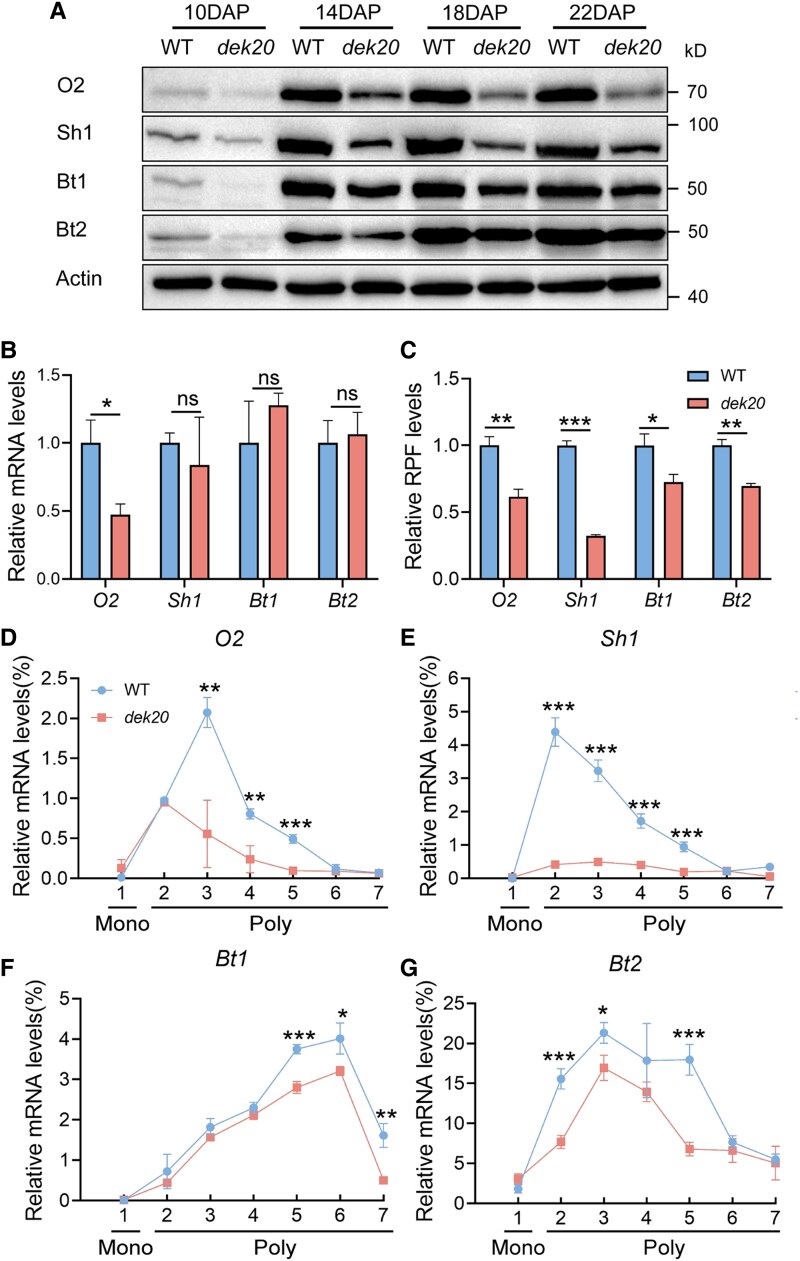
Translation analysis of critical proteins for storage compounds synthesis in *dek20*. **A)** Immunoblotting analysis of proteins for starch and protein synthesis in *dek20*. **B)** Relative mRNA level of *O2*, *Sh1*, *Bt1*, and *Bt2*. Values are means ± SD (*n* = 3, kernels from 3 independent ears; *P < 0.05; ns, no significant difference as determined by two-tailed *t*-test). **C)** Relative RPF level of *O2*, *Sh1*, *Bt1,* and *Bt2*. Values are means ± SD (*n* = 3, kernels from 3 independent ears; *P < 0.05, **P < 0.01; ***P < 0.001 as determined by two-tailed *t*-test). **D)**—**G)** Distribution of *O2*, *Sh1*, *Bt1*, and *Bt2* transcripts in sucrose gradient fractions. Actin used as control. Mono, monosome; Poly, polysome. Values are means ± SD (*n* = 3, kernels from 3 independent ears; *P < 0.05, **P < 0.01; ***P < 0.001 as determined by two-tailed *t*-test).

As shown in Fig. 1, the levels of both starch and storage proteins were significantly reduced in *dek20*. To elucidate the underlying mechanisms, we conducted a series of immunoblotting assays using antibodies against key regulators of storage protein and starch synthesis in maize, including Opaque2 (O2), Shrunken1 (Sh1), Brittle1 (Bt1), and Brittle2 (Bt2) (Fig. 11A, Supplementary Fig. S13) (Hannah and Boehlein 2017; Larkins et al. 2017). The transcription levels of *SH1*, *bt1*, and *bt2* remain unchanged, while their translation levels are significantly reduced, whereas both the transcription and translation levels of *O2* are decreased (Figs. 11, B and C). Consistent with these results, polysome profiling showed a reduced association of *O2*, *Sh1*, *Bt1*, and *Bt2* transcripts with polysomes in *dek20* kernels (Figs. 11D to G). In accordance, the protein levels of O2, Sh1, Bt1, and Bt2 were diminished in the developing kernels of *dek20* (Fig. 11A). The reduced protein level of O2 corresponded with the downregulated expression of *Zein* genes (Supplementary Fig. S14). Collectively, these results indicate that DEK20-mediated serine synthesis is crucial for storage compound synthesis by supporting the translation of key genes involved in this process.

### Serine deficiency affects cell cycle in *dek20*

**Figure 12.**
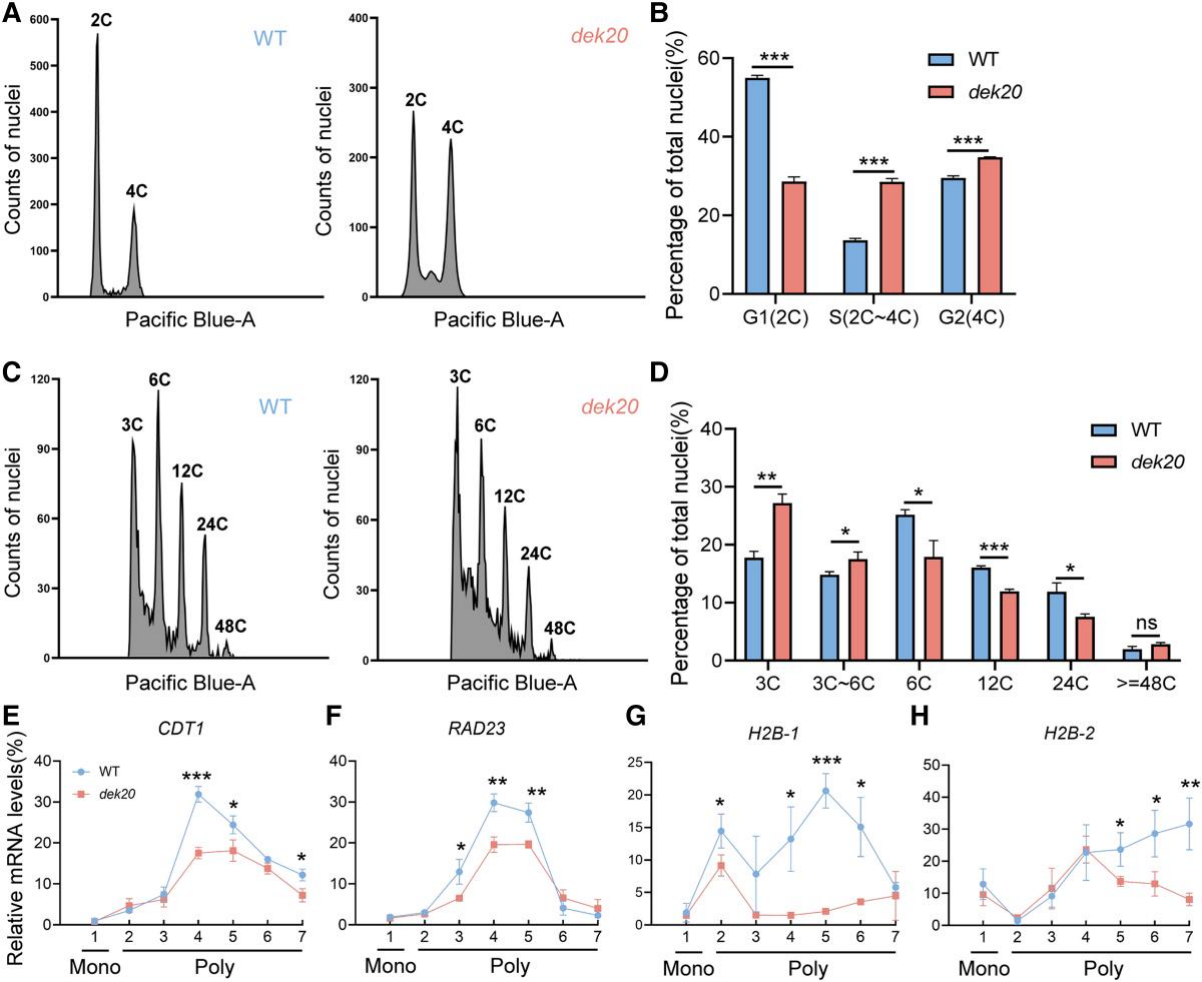
Aberrant cell cycle in *dek20* embryos and endosperms. **A)** Distribution of relative nuclear DNA contents (in relative fluorescence intensity) obtained by flow cytometry analysis of WT and *dek20* 18 DAP embryos. **B)** Histograms of DNA contents in WT and *dek20* embryos. Values are means ± SD (*n* = 3, kernels from 3 independent ears; ***P < 0.001 as determined by two-tailed *t*-test). **C)** Distribution of relative nuclear DNA contents (in relative fluorescence intensity) obtained by flow cytometry of WT and *dek20* 18 DAP endosperms. **D)** Histograms of DNA contents in WT and *dek20* endosperms. Values are means ± SD (*n* = 3, kernels from 3 independent ears; *P < 0.05, **P < 0.01, ***P < 0.001; ns, no significant difference as determined by two-tailed *t*-test). **E)** to **H)** Distribution of *CDT1*, *RAD23*, *H2B-1,* and *H2B-2* transcripts in sucrose gradient fractions. Mono, monosome; Poly, polysome. Values are means ± SD (*n* = 3, kernels from 3 independent ears; *P < 0.05, **P < 0.01, ***P < 0.001 as determined by two-tailed *t*-test).

Proper cell division is essential for kernel development. Notably, both our transcriptome and Ribo-seq analysis revealed enrichment in the biological process of the cell cycle (Supplemental Figs. 7 and 9, Supplementary Data Set 8). To assess whether the cell cycle was impaired in *dek20*, we performed flow cytometry analyses on 18 DAP embryos and endosperms (Figs. 12A to D). The results showed that the fraction of G1 phase nuclei (2C DNA content) was 29.37% in *dek20* embryos, compared to 54.97% in wild-type embryos (Fig. 12B). In contrast, the fraction of S phase nuclei (between 2C and 4C DNA content) was 29.23% in *dek20* embryos, while it was 13.6% in wild-type embryos (Fig. 12B). The fraction of G2 phase nuclei (4C DNA content) was 34.7% in *dek20* embryos, compared to 29.53% in wild-type embryos (Fig. 12B). Endoreduplication, which involves replication of the nuclear genome without cytokinesis, leads to elevated nuclear DNA content. It is a variant of the mitotic cell cycle consisting only of G1 and S phases. Flow Cytometry analysis showed that endoreduplication was repressed in *dek20* endosperms. Specifically, nuclei with C values of 3C comprised 27.19% of *dek20* endosperms, compared to 17.73% in wild-type endosperms (Fig. 12D). Additionally, nuclei with C values between 3C and 6C accounted for 17.5% of *dek20* endosperms, while only 14.8% in wild-type endosperms (Fig. 12D). Conversely, nuclei with C values of 6C or greater were less frequent in *dek20* endosperms than in wild-type endosperms (Fig. 12D). These findings suggest a block in the cell cycle, consistent with our transcriptome analysis results.

In accordance with the inhibited cell cycle in *dek20*, the translation was inhibited for chromatin licensing and DNA replication factor 1 (CDT1), RAD23, and H2B, which are essential components for DNA replication and cell cycle progression (Castellano et al. 2004; Grønbæk-Thygesen et al. 2023). RNA-seq results indicated elevated mRNA levels of *CDT1*, *RAD23*, *H2B-1*, and *H2B-2* in *dek20* (Supplementary Fig. S15A). While the translation of *RAD23* and *H2B-2* increased in *dek20*, the translation of *CDT1* and *H2B-1* remained largely unchanged (Supplementary Fig. S15B). Consequently, the translation efficiency of *RAD23*, *H2B-1*, and *H2B-2* significantly decreased in *dek20*, with *CDT1* showing a trend toward reduced efficiency (Supplementary Fig. S15C). Additionally, mRNA levels of all four genes associated with translating ribosomes were significantly lower in *dek20* than in WT (Figs. 12E to H). Notably, the protein CDT1 was enriched in serine (Supplementary Fig. S15D), suggesting that the increased ribosome pausing on serine codons may contribute to the decreased translation of these genes in *dek20*. This is consistent with findings that *CDT1* overexpression in Arabidopsis promotes endoreduplication (Castellano et al. 2004). Collectively, these data suggest that DEK20 may affect the cell cycle during kernel development by supporting the translation of key genes involved in this process.

## Discussion

### *Dek20* encodes PGDH1 and defines a predominant serine biosynthesis pathway in maize, the model plant with C4 photosynthesis

In this study, we cloned the classic maize mutant *dek20* and identified a mutation in the gene encoding PGDH, the rate-limiting enzyme of PPSB. Our results suggest that DEK20 is essential for serine synthesis and kernel development in maize. First, allelism tests between *dek20* and *dek20-2* or *dek20-3* indicated that DEK20 was required for kernel development. Second, serine content was dramatically decreased in *dek20*. Third, the *dek20* phenotype could be reversed by the external addition of serine. Finally, the DEK20(S282L) mutation released the residue His at 284, which stably bound NAD^+^, thus occupying the NAD^+^ binding domain and reducing PGDH function in subsequent rounds of serine biosynthesis. These findings suggest that serine deficiency is responsible for the defective kernel development in *dek20* and that PPSB is crucial for serine biosynthesis in maize, a model system for C4 plants with low rates of photorespiration. This is consistent with the observation that serine content is highest in the immature regions of maize leaves, where photosynthetic machinery is not yet fully developed (Wang et al. 2014). Though future functional characterization of the homologous proteins of DEK20 in other C4 plants is needed, this study suggests that PPSB may be the primary serine synthesis pathway in C4 plants. Thus, there are 2 predominant serine synthesis pathways in plants, the photorespiration-related glycolate pathway in C3 plants and the PPSB pathway in C4 plants, compared to only one pathway in bacteria or mammals.

### Structural analysis of DEK20 uncovers an important site for catalytic activity regulation

In humans, PGDH is a promising therapeutic target for cancer. Elevated PGDH activity in various cancer cells results from gene duplication, increased transcription, posttranslational modification, and allosteric regulation (Lee et al. 2024). Consequently, research focuses on identifying inhibitors of PGDH activity. Understanding the structure and active site of PGDH will aid in the development of new anti-cancer drugs. PGDH exists in at least 3 basic structural forms. DEK20 and its mammalian homologs are classified as type I PGDH, characterized by 3 major domains: the substrate binding domain, nucleotide-binding domain, and regulatory domains (Supplementary Fig. S3A). Our study resolved the structure of DEK20 and revealed that the serine residue at position 282 interacted with and stabilized the histidine residue at position 284 in the wild type. In *dek20*, this histidine residue was released and interacted stably with NAD^+^, thereby restricting PGDH enzymatic activity for subsequent serine biosynthesis (Fig. 5). These residues are conserved across different organisms (Supplementary Fig. S4) and could serve as a promising target for anti-cancer drugs.

### Serine deficiency induces the degradation of tRNA^Ser^, leading to ribosomes stalling to activate GCN2 to affect the phosphorylation of eIF2α and S6K

Our metabolomic analysis shows that free serine levels were reduced by 80% in *dek20* (Fig. 4A). Research in yeast and mammals has shown that amino acid deficiency triggers an amino acid response that increases the availability of uncharged tRNAs. These uncharged tRNAs activate the GCN2 kinase, which subsequently enhances the phosphorylation of eIF2α and inhibits the phosphorylation of S6K (Dever et al. 1992; Shu et al. 2020). Similar studies have been conducted in *Arabidopsis*, where amino acid deprivation or other stress conditions activate the *Arabidopsis* GCN2 kinase (AtGCN2), leading to the phosphorylation of eIF2α and the suppression of global protein synthesis (Lageix et al. 2008; Zhang et al. 2008; Li et al. 2013; Luna et al. 2014; Lokdarshi and von Arnim 2022). In yeast and mammals, phosphorylation of eIF2α inhibits the recycling of inactive eIF2-GDP to active eIF2-GTP, thereby limiting the formation of the ternary initiation complex, while reduced phosphorylation of S6K impairs its ability to activate downstream targets, such as ribosomal protein S6 (rpS6). Both processes ultimately result in the inhibition of translation initiation. However, the exact mechanisms underlying the inhibition of protein translation in plants remain incompletely understood (Zhigailov et al. 2020, 2022; Mancera-Martínez et al. 2021; Dasgupta et al. 2024). Here, we found that the tRNA^Ser^ level was dramatically decreased in *dek20* (Figs. 6A to H). It is believed that tRNAs become highly unstable in response to amino acid starvation (Lee and Collins 2005; Andersen and Collins 2012; Svenningsen et al. 2017), suggesting that serine deficiency may contribute to decreased tRNA^Ser^. In accordance, we indeed identified a degraded fragment of tRNA^Ser-GCT^, which is increased by 100% in *dek20* (Figs. 6, I and J).

Additionally, our Ribo-seq results indicated a reduced translation elongation speed at serine codons (Fig. 7D). GCN2 could also be activated by stalled ribosomes (Ishimura et al. 2016). Accordingly, the phosphorylation level of eIF2α was significantly increased, while S6 K phosphorylation was decreased in *dek20* endosperms (Fig. 10A). However, supplementation with 3 mm serine reduced eIF2α phosphorylation levels and increased S6 K phosphorylation in *dek20* kernels (Fig. 10B). This indicates that serine deficiency triggers the degradation of tRNA^Ser^, which slows down translation elongation speed to stall ribosomes. Then, the activation of GCN2 by stalled ribosomes further represses translation initiation.

Consistent with these findings, protein translation was repressed in *dek20*. First, polysome profiling revealed that ribosomes bound to mRNA were significantly reduced in *dek20*. Second, Ribo-seq data showed that serine deficiency led to ribosome stalling at serine codons and decreased translation efficiency. Third, the accumulation of both zein and non-zein proteins was dramatically reduced in *dek20* kernels. Fourth, the translation of proteins critical for storage compound synthesis and cell cycle transition, such as O2, Sh1, Bt1, Bt2, and CDT1 was diminished in *dek20*. The reduced levels of these proteins may account for the decreased hundred-kernel weight and inhibited cell cycles in *dek20*. Collectively, these results suggest that serine deficiency in *dek20* inhibits the initiation and elongation of protein translation. This mechanism may underlie the impaired protein synthesis and cell cycle progression observed in *PGDH1*-silenced Arabidopsis (Zimmermann et al. 2021), and the increased protein synthesis in maize transgenic lines overexpressing Arabidopsis *PGDH1* gene (Casatejada-Anchel et al. 2025).

### Local synthesis of serine is essential for kernel development

Local amino acid synthesis plays a crucial role in maize kernel development. Previous studies have shown that a mutation in *Opaque6*, which is essential for proline synthesis, results in opaque kernels and seedling lethality (Manzocchi et al. 1986). Similarly, mutations in arogenate dehydratase (ADT) or arogenate dehydrogenase (AroDH), the enzyme catalyzing the final step in phenylalanine or tyrosine synthesis, lead to severe defects in embryo and endosperm development, as well as dwarf plants (Holding et al. 2010; Ren et al. 2025). While all these mutants exhibit defective embryo and endosperm development, *dek20* stands out as the only kernel-lethal mutant. This distinction may highlight the critical role of serine in kernel development. Beyond protein translation, serine is also crucial for the biosynthesis of essential biomolecules necessary for cell proliferation, including other amino acids, nitrogenous bases, phospholipids, and sphingolipids (Ros et al. 2014). For instance, it is critical in nucleotide metabolism by maintaining folate pools and providing carbon backbones (Pacold et al. 2016; Reid et al. 2018). Consistent with this, metabolome analysis revealed differential enrichment of 9 nucleotides and their derivatives, 21 amino acids and their derivatives, 23 lipids, 14 organic acids, 6 vitamins, and 8 saccharides (Supplementary Data Set 3), suggesting that serine may also affect kernel development by influencing the synthesis of other metabolites.

Although we cannot entirely rule out the possibility that serine could be transported into the kernel, our results indicate that local synthesis of serine is essential for kernel development. On one hand, both the embryo and endosperm undergo rapid cell division during early development and extensive storage of protein synthesis during the later filling stage, both of which require significant amounts of metabolites such as serine and its derivatives. However, several factors, including the limited sink capacity during early kernel development, the absence of vascular tissue in meristematic regions, the slow rate of membrane transport, and the restriction of the glycolate pathway to daylight hours, may contribute to an insufficient external supply of serine to meet these metabolic demands. On the other hand, diverting glycolytic flux into de novo serine biosynthesis via PPSB has several critical biological implications. For example, the PPSB contributes to the provision of α-ketoglutaric acid through anaplerotic reactions, channeling glutamine-derived carbon into the TCA cycle to counterbalance biosynthetic efflux. A reduction in PGDH function would result in a significant loss of α-ketoglutaric acid (Possemato et al. 2011). Though the content of α-Ketoglutaric acid was unaffected in 14 DAP *dek20* kernels, it may still represent another possibility. These findings align with previous research in Arabidopsis. It was suggested that during early embryogenesis, many nutrients are supplied to the embryo by the endosperm and maternal tissues, whereas, in later stages, the embryo begins synthesizing most metabolites autonomously (Cascales-Miñana et al. 2013). Failure to establish the PPSB in related mutants leads to arrested embryo development (Benstein et al. 2013; Cascales-Miñana et al. 2013; Toujani et al. 2013).

In summary, DEK20, the rate-limiting enzyme in the phosphorylated pathway of serine biosynthesis (PPSB) is essential for serine synthesis in maize, the model plant with C4 photosynthesis. Without DEK20, serine content decreases dramatically. The resultant serine deficiency triggers the degradation of tRNA^Ser^, leading to stalled ribosomes to activate GCN2, which further affects the phosphorylation of eIF2α and S6K1 to repress general protein synthesis and cell cycle transition. This disruption affects the synthesis of zein, non-zein proteins, starch metabolism, and cell cycle-related proteins, leading to the lethal embryo and reduced endosperm in *dek20*, linking amino acid metabolic processes directly to developmental outcomes in plants.

## Materials and methods

### Plant materials

The maize (*Zea mays*) *dek20* (*dek20*-*N1392A*) mutant, generated by EMS, was obtained from the Maize Genetics Cooperation Stock Center. The *dek20* stock was backcrossed into the B73 inbred line 3 times, and kernels were collected from a self-pollinated heterozygous *dek20*/+ ear with a predominantly B73 genetic background. The other 2 EMS-induced *Dek20* mutants were obtained from the Maize EMS-induced Mutant Database (mutant IDs: *EMS3*-*02265d* and *EMS3*-*1701bc*). Maize plants were planted in the experimental fields in Piscataway, New Jersey, USA, or Qingdao, Shandong, China, for summer nursery, or in Molokai, Hawaii, USA, or Ledong, Hainan, China, for winter nursery.

### Confocal microscopy analysis

The coding sequence (CDS) of *Dek20* was cloned into the transient expression vector pYBA1132-GFP (provided by Prof. Zhongyi Wu, BAAFS), under the control of the CaMV 35S promoter. The resulting fusion construct was introduced into maize protoplasts via polyethylene glycol (PEG)/calcium-mediated transformation. Following a 12-hour incubation in the dark, fluorescence signals from the fusion proteins were observed using a Zeiss LSM900 confocal fluorescence microscope (Zeiss, Germany). GFP fluorescence was detected with an excitation wavelength of 488 nm, while chlorophyll autofluorescence was excited at 561 nm.

### Quantification of starch and protein content

Starch content was determined using the Megazyme Total Starch Assay Kit (KTSTA-50A) according to the manufacturer's protocols. The starch contents for each sample and the D-glucose control were evaluated at an absorbance of 510 nm. Zein and non-zein proteins were extracted according to published methods (Zhang et al. 2019). Protein quantification was performed using the BCA Protein Assay Kit (Epizyme, ZJ102). All measurements were performed at least 3 times.

### Light microscopy and transmission electron microscopy analysis

For light microscopy, immature WT and *dek20* kernels at 10DAP, 14DAP, and 18DAP were collected from the same ear, cut longitudinally, fixed in Formalin-Aceto-Alcohol solution (5 ml 38% formaldehyde, 5 ml glacial acetic acid, and 90 ml 70% ethanol) for 3 days, embedded in paraffin, sliced (Leica, RM2265), stained with 0.2% toluidine blue, and observed with a Leica MZ10 F microscope.

For transmission electron microscopy, 2–5 mm slices of developing WT and *dek20* endosperm tissues from the same ear were fixed overnight in 0.1 m sodium cacodylate buffer (pH, 7.4) with 2.5% glutaraldehyde, washed with PBS (pH, 7.2), dehydrated in an ethanol gradient, transferred to propylene oxide, and infiltrated with acrylic resin. Ultrathin sections were cut (Leica, EM UC7) and imaged with a Zeiss Crossbeam 550 microscope.

### Expression and purification of DEK20 protein

The DEK20, DEK20(S282L), and DEK20(S282L H284A) clones were transformed into *E. coli* BL21(DE3) and induced with 0.5 mm Isopropyl β-D-Thiogalactoside at 16 ℃ overnight. Protein purification was performed using the His-tag Protein Purification Kit (Beyotime, P2226). The eluted fractions were concentrated and applied to 120 ml Superdex 200 Increase 10/300 GL size-exclusion column (GE Healthcare) equilibrated with gel filtration buffer (10 mm Tris-HCl of pH 7.6, 100 mm NaCl, 0.1 mm EDTA, and 3 mm DTT). The fractions containing target proteins were concentrated and stored in aliquots at −80 °C. Primer sequences are listed in Supplemental Data 11.

### Crystallization, structure determination, and refinement

About 16 mg/ml DEK20^WT^ protein samples were crystallized in the solution of 0.3 m sodium nitrate, 0.3 m sodium phosphate dibasic, 0.3 m ammonium sulfate, 1 m sodium HEPES, 1 m MOPS (acid) of pH 7.5, 40% (v/v) glycerol, 20% (w/v) PEG 4000; 6 mg/ml DEK20^S282L^ protein samples were crystallized in the solution of 0.2 m sodium formate, 0.2 m ammonium acetate, 0.2 m sodium citrate tribasic dihydrate, 0.2 m potassium sodium tartrate tetrahydrate, 0.2 m sodium oxamate, 1 m Tris (base), BICINE of pH 8.5, 40% (v/v) ethylene glycol, 20% (w/v) PEG 8000. The X-ray diffraction data were collected on beamlines BL10U2 at the Shanghai Synchrotron Radiation Facility (SSRF). HKL2000 program was used to process the data.

The apo form DEK20^WT^ and DEK20^S282L^ structures were solved by molecular replacement (MR) method with the Phaser program embedded in the CCP4i suite. The model generated by the AlphaFold3 program serves as the search model. The 2Fo–Fc and Fo–Fc electron density maps were regularly calculated and used as a guide for building the missing amino acids using COOT. The 3-PGA and NAD^+^ molecules were built manually using COOT. The final refinement was done using the phenix.refine program of the Phenix suit.

### DEK20 enzymic activity assay

PGDH activity was determined according to the published methods with minor modifications (Benstein et al. 2013). The assay was performed in a total volume of 200 *μ*l containing 10 *μ*l of purified enzyme, 200 mm Tris (pH 8.1), 25 mm EDTA, 0.1 mm DTT, 5 mm hydrazine sulfate, 0.5 mm NAD^+^, and variable concentrations of 3-phosphoglycerate (Sigma-Aldrich, P8877). The reaction was started by the addition of the purified enzyme. After 60 min of reaction at 30℃, the light absorption value of NADH was measured to calculate enzyme activity.

### Microscale thermophoresis assay

The recombinant proteins His-DEK20, His-DEK20(S282L), and His-DEK20(S282L H284A) were labeled with the Monolith Protein Labeling Kit RED-NHS 2nd Generation (NanoTemper, MO-L018). The microscale thermophoresis assays were conducted using a Monolith NT.115 (NanoTemper) machine. Each protein was labeled 3 times for 3 independent tests. All data were analyzed using the MO.Affinity Analysis version 2.3 software.

### Western blot

PVDF Membranes were blocked with 5% (w/v) low-fat milk and then blotted with anti-Actin (1:5000, Abclonal, AC009), anti-eIF2α (1:500, Agrisera, AS204371), anti-eIF2α(S51) (1:1000, Abclonal, AP0692), anti-Sh1 (1:3000, Orizymes, PAB191113), anti-Bt1 (1:3000, Orizymes, PAB200116), anti-O2, and anti-Bt2 (1:5000). For detection of the protein level of S6 K, PVDF membranes were blocked with 3% (w/v) BSA for phosphorylated S6 K or 5% (w/v) low-fat milk for S6 K in TBS with 0.1% Tween-20. Anti-S6 K (phospho T449, 1:5000, Abcam, ab207399) or anti-S6 K (1:1000, Agrisera, AS121855) were used to detect the phosphorylation of S6 K.

### Kernel culture in vitro

Developing kernel in vitro culture followed a modified version of a previously described method (Wang et al. 2022). Ears were harvested at 4 DAP, husks removed, sterilized with 95% alcohol, dried, and immersed in 5% bleach for 5 min in a laminar-flow hood. The ears were dissected into three-row blocks, each containing 3 kernels, and placed in 100 × 25-mm plastic dishes with Murashige and Skoog medium (supplemented with 1 mg L⁻¹ 2,4-D, 15% sucrose, 5.5 g L⁻¹ agar, and 10 mg L⁻¹ Streptomycin sulfate). Kernels were cultured in the dark at 28 °C for 12 days.

### Flow cytometry

WT or *dek20* mutant kernels were collected from the same ear as a replicate. Three biological replicates from 3 independent ears were used. Endosperms and embryos were finely chopped with a sharp razor blade in Galbraith's lysis buffer. The resulting mixture was filtered through a 42-μm nylon filter to eliminate cell debris, and the suspension containing nuclei was immediately measured using a Facscan (Becton Dickinson) laser flow cytometer equipped with an argon-ion laser tuned to a wavelength of 448 nm. For each sample, a total of 5,000 particles were collected and analyzed using FlowJo software (FlowJo, Ashland, OR).

### Metabolomics analysis

Fourteen DAP WT or *dek20* kernels were freeze-dried using a vacuum freeze-dryer (Scientz-100F). Kernels from 3 independent ears were used as 3 biological replicates. The freeze-dried samples were crushed using a mixer mill (Retsch, MM 400) with a zirconia bead for 1.5 min at 30 Hz; 50 mg of the lyophilized powder was dissolved in 1.2 mL of 70% methanol solution. Following centrifugation at 13,000 g for 3 min, the extracts were filtered (SCAA-104, 0.22-*μ*m pore size; ANPEL). Then, the extracts were analyzed using a UPLC-ESI-MS/MS system (UPLC, ExionLC Ad; MS, Applied Biosystems 4500 Q TRAP). Differential metabolites were determined based on Variable Importance in Projection (VIP ≥ 1) and Foldchange ≥ 2 or Foldchange ≤ 0.5.

### Northern blot

To compare tRNA levels via northern blot, 2 to 5 mg of total RNA was separated on an 8% denaturing PAGE gel and transferred to a Hybond-N + nylon membrane (GE Healthcare, RPN303B) using a semi-dry transfer system (Bio-Rad) in 0.5 × TBE buffer at 300 mA for 30 min. The membrane was UV crosslinked (UVP Laboratory Products) and hybridized with biotin-labeled tRNA probes, and then developed using the Chemiluminescent Nucleic Acid Detection Module kit (Thermo Fisher, 89880). Biotin-labeled probes sequences are listed in Supplementary Data Set 11.

### tRNA RT-qPCR

The relative levels of tRNA^Ser-TGA^ and tRNA^Ser-GCT^ were quantified based on published protocols with minor modifications (Pavlova et al. 2020); 14 DAP WT and *dek20* kernels were flash-frozen in liquid nitrogen, ground to a fine powder, and lysed in cold TRIzol reagent (Transgen, ET101) on ice. Lysates were extracted with chloroform (5:1, v/v), centrifuged at 18,600 g, and RNA was precipitated overnight with 2.7 volumes of cold ethanol and 30 mg of GlycoBlue Coprecipitant (ThermoFisher, AM9515). The RNA pellet was resuspended in 0.3 m sodium acetate buffer (pH, 4.5) containing 10 mm EDTA and reprecipitated. The following day, RNA was resuspended in 10 mm sodium acetate buffer (pH, 4.5) with 1 mm EDTA. Yeast tRNA^Phe^ (Sigma-Aldrich, R4018) was added as an internal control, followed by ethanol precipitation. The pellet was resuspended in 50 mm Tris buffer (pH, 9.0) and incubated at 37 °C for 50 min for deacylation, quenched with acetate buffer, and reprecipitated. RNA was then resuspended in RNase-free water and ligated to a 5′-adenylated DNA adaptor (5′/5rApp/TGGAATTCTCGGGTGCCAAGG/3ddC/-3′) using truncated KQ mutant T4 RNA ligase 2 (New England Biolabs, M0373) for 3 h at room temperature. After ligation, RNA was again resuspended in RNase-free water. Reverse transcription was performed using SuperScript IV reverse transcriptase (Mei5 Biotechnology, MF011) following the manufacturer's protocol. The resulting cDNA was analyzed by qPCR using tRNA isodecoder-specific primers (sequences provided in Supplemental Data 11). Primers were designed based on reference tRNA sequences from the GtRNAdb database (http://gtrnadb.ucsc.edu/) (Chan and Lowe 2016). Ct values obtained with yeast tRNA^Phe^ primers were used for normalization and subtracted from Ct values of the corresponding tRNA isodecoders.

### Ribosome profiling

Fourteen DAP WT and *dek20* kernels were frozen in liquid nitrogen, ground into powder, and dissolved in 400 *µ*L of lysis buffer. Kernels from 3 independent ears were used as 3 biological replicates. Ribosome profiling was performed and sequenced on an Illumina NovaSeq X Plus by Gene Denovo Biotechnology Co. (Guangzhou, China).

Raw reads were trimmed, filtered and aligned to the version 5 B73 reference genome. Only uniquely mapped reads were analyzed. BAM files were used to calculate metrics like ribosome-protected fragment (RPF) length and offset detection via a custom Python pipeline. In-frame reads of 29–33 nucleotides were used for downstream analysis. RPF abundance was calculated for each gene, and differential gene expression was assessed with DESeq2. Genes with more than 10 reads were used to calculate pausing scores. Translation efficiency (TE) was determined by dividing RPF abundance by mRNA levels, with differential TE analysis performed using deltaTE.

### Polysome profiling

Polysome profiling was performed according to the published method (He et al. 2024). Kernels from 3 independent ears were used as 3 biological replicates. To compare the global translation status across samples, the levels of total RNA, monosome, and polysome were quantified using ImageJ based on the absorbance data for each sample. Subsequently, the ratios of polysome to monosome (P/M) were calculated. To assess the translation of specific mRNA, RNAs were extracted from both the “Input” samples and gradient fractions. Values from each fraction were normalized to the “Input” sample and presented as a percentage of the input. Primer sequences are listed in Supplementary Data Set 11.

### Accession numbers

Sequence data from this article can be found in the GenBank/EMBL data libraries under accession numbers: *Dek20*, Zm00001d002051; *O2*, Zm00001d018971; *Sh1*, Zm00001d045042; *Bt1*, Zm00001d015746; *Bt2*, Zm00001d050032; *15-kD β-zein*, Zm00001d035760; *16-kD γ-zein*, Zm00001d005793; *19-kD-z1A α-zein*, Zm00001d048847; *19-kD-z1B α-zein*, Zm00001d019155; *19-kD-z1D α-zein*, Zm00001d030855; *22-kD-z1C α-zein*, Zm00001d048809; *27-kD γ-zein*, Zm00001d020592; *50-kD γ-zein*, Zm00001d020591; *CDT1*, Zm00001d002056; *RAD23*, Zm00001d053738; *H2B-1*, Zm00001d005789; *H2B-2*, Zm00001eb124070.

## Supplementary Material

koaf126_Supplementary_Data

## Data Availability

The raw RNA-seq and Ribo-seq data from this study have been deposited at the China National Center for Bioinformation under accession numbers CRA019220 and CRA019221. The protein structure is deposited into the Protein Data Bank (PDB) under accession numbers 9JCN and 9JCM.

## Supplementary data

The following materials are available in the online version of this article.

**Supplementary Figure S1.** Chi-square tests of the defective kernel phenotype in *dek20/+* selfed ears.

**Supplementary Figure S2.** Histological analysis of developing WT and *dek20* kernels.

**Supplementary Figure S3.** Phylogenetic analysis of *Dek20*.

**Supplementary Figure S4.** Amino acid sequence alignment of DEK20 and its homologs from representative species.

**Supplementary Figure S5.** Expression pattern of *Dek20* homologous genes.

**Supplementary Figure S6.** Microscale thermophoresis analysis of the affinity to NAD^+^ for DEK20, DEK20 (S282L), and DEK20(S282L H284A) proteins.

**Supplementary Figure S7.** Transcriptome analysis of *dek20*.

**Supplementary Figure S8.** Ribosome profiling sequencing statistics of WT and *dek20* kernels.

**Supplementary Figure S9.** Translatome analysis of *dek20*.

**Supplementary Figure S10.** Plots of pausing scores for individual codons at the ribosomal E and P sites in *dek20* versus WT kernels.

**Supplementary Figure S11.** eIF2α And S6K1 phosphorylation analysis.

**Supplementary Figure S12.** eIF2α and S6K1 phosphorylation analysis after cultivated in medium supplemented with 3 mm L-serine or 3 mm L-tyrosine.

**Supplementary Figure S13.** Immunoblotting analysis of proteins for starch and protein synthesis in *dek20*.

**Supplementary Figure S14.** Expression analysis of *Zein* genes.

**Supplementary Figure S15.** Translation analysis of cell cycle-related genes.

**Supplementary Data Set 1.** The candidate mutations of *dek20*

**Supplementary Data Set 2.** Chi-square tests for selfed and allelism-crossed ears between *dek20* and *dek20-2* or *dek20-3*

**Supplementary Data Set 3.** List of differential metabolites in *dek20* versus WT

**Supplementary Data Set 4.** Statistics of DEK20^WT^ and DEK20^S282L^ protein structure determination and refinement

**Supplementary Data Set 5.** List of differentially expressed genes in *dek20* versus WT

**Supplementary Data Set 6.** List of differentially translated genes in *dek20* versus WT

**Supplementary Data Set 7.** GO Enrichment analysis of the transcriptome, translatome, translational efficiency and nine quadrants analysis

**Supplementary Data Set 8.** List of differentially translated efficiency genes in *dek20* versus WT

**Supplementary Data Set 9.** List of genes from the nine quadrants analysis

**Supplementary Data Set 10.** Expression of the genes in the GO term of serine family amino acid metabolic pathway

**Supplementary Data Set 11.** Primers and probes used in this paper

## Dive Curated Terms

The following phenotypic, genotypic, and functional terms are of significance to the work described in this paper:

PGDH Gramene: AT1G17745
PGDH Araport: AT1G17745

PSAT Gramene: AT4G35630
PSAT Araport: AT4G35630

Psp Gramene: AT1G18640
Psp Araport: AT1G18640

Bt1 Gramene: AT5G63160
Bt1 Araport: AT5G63160

NAD CHEBI: CHEBI:13389

serine CHEBI: CHEBI:17822

RAD23 Gramene: AT1G79650
RAD23 Araport: AT1G79650

BT2 Gramene: Zm00001eb176800
BT2 Araport: Zm00001eb176800

GCN2 Gramene: At3g59410
GCN2 Araport: At3g59410

PGDH1 Gramene: At4g34200
PGDH1 Araport: At4g34200

## References

[koaf126-B1] Amelio I, Cutruzzolá F, Antonov A, Agostini M, Melino G. Serine and glycine metabolism in cancer. Trends Biochem Sci. 2014:39(4):191–198. 10.1016/j.tibs.2014.02.004 24657017 PMC3989988

[koaf126-B2] Andersen KL, Collins K. Several RNase T2 enzymes function in induced tRNA and rRNA turnover in the ciliate *Tetrahymena*. Mol Biol Cell. 2012:23(1):36–44. 10.1091/mbc.e11-08-0689 22049026 PMC3248902

[koaf126-B3] Bauwe H, Hagemann M, Fernie AR. Photorespiration: players, partners and origin. Trends Plant Sci. 2010:15(6):330–336. 10.1016/j.tplants.2010.03.006 20403720

[koaf126-B4] Benstein RM, Ludewig K, Wulfert S, Wittek S, Gigolashvili T, Frerigmann H, Gierth M, Flügge U-I, Krueger S. *Arabidopsis* phosphoglycerate dehydrogenase1 of the phosphoserine pathway is essential for development and required for ammonium assimilation and tryptophan biosynthesis. Plant Cell. 2013:25(12):5011–5029. 10.1105/tpc.113.118992 24368794 PMC3904002

[koaf126-B5] Bukowski R, Guo X, Lu Y, Zou C, He B, Rong Z, Wang B, Xu D, Yang B, Xie C, et al Construction of the third-generation *Zea mays* haplotype map. GigaScience. 2018:7(4):1–12. 10.1093/gigascience/gix134 PMC5890452 29300887

[koaf126-B6] Casatejada-Anchel R, Torres-Moncho A, Anoman AD, Budhagatapalli N, Pérez-Lorences E, Alcántara-Enguídanos A, Rosa-Téllez S, de Souza LP, Kumlehn J, Fernie AR, et al Metabolic engineering of the serine/glycine network as a means to improve the nitrogen content of crops. Plant Biotechnol J. 2025:23(1):268–280. 10.1111/pbi.14495 39450589 PMC11672731

[koaf126-B7] Cascales-Miñana B, Muñoz-Bertomeu J, Flores-Tornero M, Anoman AD, Pertusa J, Alaiz M, Osorio S, Fernie AR, Segura J, Ros R. The phosphorylated pathway of serine biosynthesis is essential both for male gametophyte and embryo development and for root growth in *Arabidopsis*. Plant Cell. 2013:25(6):2084–2101. 10.1105/tpc.113.112359 23771893 PMC3723614

[koaf126-B8] Castellano MdM, Boniotti MB, Caro E, Schnittger A, Gutierrez C. DNA replication licensing affects cell proliferation or endoreplication in a cell type–specific manner. Plant Cell. 2004:16(9):2380–2393. 10.1105/tpc.104.022400 15316110 PMC520940

[koaf126-B9] Chan PP, Lowe TM. GtRNAdb 2.0: an expanded database of transfer RNA genes identified in complete and draft genomes. Nucleic Acids Res. 2016:44(D1):D184–D189. 10.1093/nar/gkv1309 26673694 PMC4702915

[koaf126-B10] Chaneton B, Hillmann P, Zheng L, Martin ACL, Maddocks ODK, Chokkathukalam A, Coyle JE, Jankevics A, Holding FP, Vousden KH, et al Serine is a natural ligand and allosteric activator of pyruvate kinase M2. Nature. 2012:491(7424):458–462. 10.1038/nature11540 23064226 PMC3894725

[koaf126-B11] Chen J, Zeng B, Zhang M, Xie S, Wang G, Hauck A, Lai J. Dynamic transcriptome landscape of maize embryo and endosperm development. Plant Physiol. 2014:166(1):252–264. 10.1104/pp.114.240689 25037214 PMC4149711

[koaf126-B12] Dasgupta A, Urquidi Camacho RA, Enganti R, Cho SK, Tucker LL, Torreverde JS, Abraham PE, von Arnim AG. A phosphorylation-deficient ribosomal protein eS6 is largely functional in *Arabidopsis thaliana*, rescuing mutant defects from global translation and gene expression to photosynthesis and growth. Plant Direct. 2024:8(1):e566. 10.1002/pld3.566 38250458 PMC10799217

[koaf126-B13] Dever TE, Feng L, Wek RC, Cigan AM, Donahue TF, Hinnebusch AG. Phosphorylation of initiation factor 2 alpha by protein kinase GCN2 mediates gene-specific translational control of GCN4 in yeast. Cell. 1992:68(3):585–596. 10.1016/0092-8674(92)90193-G 1739968

[koaf126-B14] Dey S, Hu Z, Xu XL, Sacchettini JC, Grant GA. D-3-phosphoglycerate dehydrogenase from *Mycobacterium tuberculosis* is a link between the *Escherichia coli* and mammalian enzymes. J Biol Chem. 2005:280(15):14884–14891. 10.1074/jbc.M414488200 15668250

[koaf126-B15] Dong J, Tu M, Feng Y, Zdepski A, Ge F, Kumar D, Slovin JP, Messing J. Candidate gene identification of existing or induced mutations with pipelines applicable to large genomes. Plant J. 2019:97(4):673–682. 10.1111/tpj.14153 30417446

[koaf126-B16] Douce R, Bourguignon J, Neuburger M, Rébeillé F. The glycine decarboxylase system: a fascinating complex. Trends Plant Sci. 2001:6(4):167–176. 10.1016/S1360-1385(01)01892-1 11286922

[koaf126-B17] Dunn JG, Foo CK, Belletier NG, Gavis ER, Weissman JS. Ribosome profiling reveals pervasive and regulated stop codon readthrough in *Drosophila melanogaster*. Elife. 2013:2:e01179. 10.7554/eLife.01179 24302569 PMC3840789

[koaf126-B18] Fu Y, Xiao W, Tian L, Guo L, Ma G, Ji C, Huang Y, Wang H, Wu X, Yang T, et al Spatial transcriptomics uncover sucrose post-phloem transport during maize kernel development. Nat Commun. 2023:14(1):7191. 10.1038/s41467-023-43006-7 37938556 PMC10632454

[koaf126-B19] Gaufichon L, Marmagne A, Belcram K, Yoneyama T, Sakakibara Y, Hase T, Grandjean O, Clément G, Citerne S, Boutet-Mercey S, et al ASN1-encoded asparagine synthetase in floral organs contributes to nitrogen filling in *Arabidopsis* seeds. Plant J. 2017:91(3):371–393. 10.1111/tpj.13567 28390103

[koaf126-B20] Grønbæk-Thygesen M, Kampmeyer C, Hofmann K, Hartmann-Petersen R. The moonlighting of RAD23 in DNA repair and protein degradation. Biochim Biophys Acta Gene Regul Mech. 2023:1866(2):194925. 10.1016/j.bbagrm.2023.194925 36863450

[koaf126-B21] Guo N, Zhang S, Gu M, Xu G. Function, transport, and regulation of amino acids: what is missing in rice? Crop J. 2021:9(3):530–542. 10.1016/j.cj.2021.04.002

[koaf126-B22] Hannah LC, Boehlein S. Starch biosynthesis in maize endosperm. In: Larkins BA, editors. Maize kernel development. Oxfordshire, UK: CAB International; 2017. p. 149–159.

[koaf126-B23] Hay N, Sonenberg N. Upstream and downstream of mTOR. Genes Dev. 2004:18(16):1926–1945. 10.1101/gad.1212704 15314020

[koaf126-B24] He R, Lv Z, Li Y, Ren S, Cao J, Zhu J, Zhang X, Wu H, Wan L, Tang J, et al tRNA-m^(1)^A methylation controls the infection of *Magnaporthe oryzae* by supporting ergosterol biosynthesis. Dev Cell. 2024:59(22):2931–2946.e7. 10.1016/j.devcel.2024.08.002 39191251

[koaf126-B25] Holding DR, Meeley RB, Hazebroek J, Selinger D, Gruis F, Jung R, Larkins BA. Identification and characterization of the maize *arogenate dehydrogenase* gene family. J Exp Bot. 2010:61(13):3663–3673. 10.1093/jxb/erq179 20558569 PMC2921203

[koaf126-B26] Huang Y, Wang H, Zhu Y, Huang X, Li S, Wu X, Zhao Y, Bao Z, Qin L, Jin Y, et al THP9 enhances seed protein content and nitrogen-use efficiency in maize. Nature. 2022:612(7939):292–300. 10.1038/s41586-022-05441-2 36385527

[koaf126-B27] Ingolia NT, Ghaemmaghami S, Newman JR, Weissman JS. Genome-wide analysis in vivo of translation with nucleotide resolution using ribosome profiling. Science. 2009:324(5924):218–223. 10.1126/science.1168978 19213877 PMC2746483

[koaf126-B28] Ishimura R, Nagy G, Dotu I, Chuang JH, Ackerman SL. Activation of GCN2 kinase by ribosome stalling links translation elongation with translation initiation. Elife. 2016:5:e14295. 10.7554/eLife.14295 27085088 PMC4917338

[koaf126-B29] Jöchl C, Rederstorff M, Hertel J, Stadler PF, Hofacker IL, Schrettl M, Haas H, Hüttenhofer A. Small ncRNA transcriptome analysis from *Aspergillus fumigatus* suggests a novel mechanism for regulation of protein synthesis. Nucleic Acids Res. 2008:36(8):2677–2689. 10.1093/nar/gkn123 18346967 PMC2377427

[koaf126-B30] Kalhan SC, Hanson RW. Resurgence of serine: an often neglected but indispensable amino acid. J Biol Chem. 2012:287(24):19786–19791. 10.1074/jbc.R112.357194 22566694 PMC3370164

[koaf126-B31] Kawade K, Tabeta H, Ferjani A, Hirai MY. The roles of functional amino acids in plant growth and development. Plant Cell Physiol. 2023:64(12):1482–1493. 10.1093/pcp/pcad071 37489637

[koaf126-B32] Kilberg MS, Pan Y-X, Chen H, Leung-Pineda V. Nutritional control of gene expression: how mammalian cells respond to amino acid limitation. Annu Rev Nutr. 2005:25(1):59–85. 10.1146/annurev.nutr.24.012003.132145 16011459 PMC3600373

[koaf126-B33] Lageix S, Lanet E, Pouch-Pélissier MN, Espagnol MC, Robaglia C, Deragon JM, Pélissier T. Arabidopsis eIF2alpha kinase GCN2 is essential for growth in stress conditions and is activated by wounding. BMC Plant Biol. 2008:8(1):134. 10.1186/1471-2229-8-134 19108716 PMC2639386

[koaf126-B34] Larkins BA, Wu Y, Song R, Messing J. Maize seed storage protein. In: Larkins BA, editor. In Maize kernel development. Oxfordshire, UK: CAB International; 2017. p. 175–189.

[koaf126-B35] Lee CM, Hwang Y, Kim M, Park Y-C, Kim H, Fang S. PHGDH: a novel therapeutic target in cancer. Exp Mol Med. 2024:56(7):1513–1522. 10.1038/s12276-024-01268-1 38945960 PMC11297271

[koaf126-B36] Lee S, Park J, Lee J, Shin D, Marmagne A, Lim PO, Masclaux-Daubresse C, An G, Nam HG. *OsASN1* overexpression in rice increases grain protein content and yield under nitrogen-limiting conditions. Plant Cell Physiol. 2020:61(7):1309–1320. 10.1093/pcp/pcaa060 32384162 PMC7377344

[koaf126-B37] Lee SR, Collins K. Starvation-induced cleavage of the tRNA anticodon loop in *Tetrahymena thermophila*. J Biol Chem. 2005:280(52):42744–42749. 10.1074/jbc.M510356200 16272149

[koaf126-B38] Li M-W, AuYeung W-K, Lam H-M. The GCN2 homologue in *Arabidopsis thaliana* interacts with uncharged tRNA and uses Arabidopsis eIF2α molecules as direct substrates. Plant Biology. 2013:15(1):13–18. 10.1111/j.1438-8677.2012.00606.x 22672016

[koaf126-B39] Locasale JW, Grassian AR, Melman T, Lyssiotis CA, Mattaini KR, Bass AJ, Heffron G, Metallo CM, Muranen T, Sharfi H, et al Phosphoglycerate dehydrogenase diverts glycolytic flux and contributes to oncogenesis. Nat Genet. 2011:43(9):869–874. 10.1038/ng.890 21804546 PMC3677549

[koaf126-B40] Lokdarshi A, von Arnim AG. Review: emerging roles of the signaling network of the protein kinase GCN2 in the plant stress response. Plant Sci. 2022:320:111280. 10.1016/j.plantsci.2022.111280 35643606 PMC9197246

[koaf126-B41] Lu X, Liu J, Ren W, Yang Q, Chai Z, Chen R, Wang L, Zhao J, Lang Z, Wang H, et al Gene-indexed mutations in maize. Mol Plant. 2018:11(3):496–504. 10.1016/j.molp.2017.11.013 29223623

[koaf126-B42] Luna E, van Hulten M, Zhang Y, Berkowitz O, López A, Pétriacq P, Sellwood MA, Chen B, Burrell M, van de Meene A, et al Plant perception of β-aminobutyric acid is mediated by an aspartyl-tRNA synthetase. Nat Chem Biol. 2014:10(6):450–456. 10.1038/nchembio.1520 24776930 PMC4028204

[koaf126-B43] Mancera-Martínez E, Dong Y, Makarian J, Srour O, Thiébeauld O, Jamsheer M, Chicher J, Hammann P, Schepetilnikov M, Ryabova LA. Phosphorylation of a reinitiation supporting protein, RISP, determines its function in translation reinitiation. Nucleic Acids Res. 2021:49(12):6908–6924. 10.1093/nar/gkab501 34133725 PMC8266674

[koaf126-B44] Manzocchi L, Tonelli C, Gavazzi G, Di Fonzo N, Soave C. Genetic relationship between o6 and pro-1 mutants in maize. Theor Appl Genet. 1986:72(6):778–781. 10.1007/BF00266544 24248199

[koaf126-B45] Maurino VG, Peterhansel C. Photorespiration: current status and approaches for metabolic engineering. Curr Opin Plant Biol. 2010:13(3):249–256. 10.1016/j.pbi.2010.01.006 20185358

[koaf126-B46] Megel C, Hummel G, Lalande S, Ubrig E, Cognat V, Morelle G, Salinas-Giegé T, Duchêne A-M, Maréchal-Drouard L. Plant RNases T2, but not Dicer-like proteins, are major players of tRNA-derived fragments biogenesis. Nucleic Acids Res. 2019:47(2):941–952. 10.1093/nar/gky1156 30462257 PMC6344867

[koaf126-B47] Michard E, Lima PT, Borges F, Silva AC, Portes MT, Carvalho JE, Gilliham M, Liu L-H, Obermeyer G, Feijó JA. Glutamate receptor like genes form Ca2 + channels in pollen tubes and are regulated by pistil D-serine. Science. 2011:332(6028):434–437. 10.1126/science.1201101 21415319

[koaf126-B48] Misra J, Carlson KR, Spandau DF, Wek RC. Multiple mechanisms activate GCN2 eIF2 kinase in response to diverse stress conditions. Nucleic Acids Res. 2024:52(4):1830–1846. 10.1093/nar/gkae006 38281137 PMC10899773

[koaf126-B49] Mothet JP, Parent AT, Wolosker H, Brady RO Jr., Linden DJ, Ferris CD, Rogawski MA, Snyder SH. D-serine is an endogenous ligand for the glycine site of the N-methyl-D-aspartate receptor. Proc Natl Acad Sci U S A. 2000:97(9):4926–4931. 10.1073/pnas.97.9.4926 10781100 PMC18334

[koaf126-B50] Mullarky E, Lucki NC, Beheshti Zavareh R, Anglin JL, Gomes AP, Nicolay BN, Wong JC, Christen S, Takahashi H, Singh PK, et al Identification of a small molecule inhibitor of 3-phosphoglycerate dehydrogenase to target serine biosynthesis in cancers. Proc Natl Acad Sci U S A. 2016:113(7):1778–1783. 10.1073/pnas.1521548113 26831078 PMC4763784

[koaf126-B51] Neuffer G, Sheridan WF. Defective kernel mutants of maize. 1. genetic and lethality studies. Genetics. 1980:95(4):929–944. 10.1093/genetics/95.4.929 17249053 PMC1214277

[koaf126-B52] Pacold ME, Brimacombe KR, Chan SH, Rohde JM, Lewis CA, Swier LJYM, Possemato R, Chen WW, Sullivan LB, Fiske BP, et al A PHGDH inhibitor reveals coordination of serine synthesis and one-carbon unit fate. Nat Chem Biol. 2016:12(6):452–458. 10.1038/nchembio.2070 27110680 PMC4871733

[koaf126-B53] Pavlova NN, King B, Josselsohn RH, Violante S, Macera VL, Vardhana SA, Cross JR, Thompson CB. Translation in amino-acid-poor environments is limited by tRNA^(Gln)^ charging. Elife. 2020:9:e62307. 10.7554/eLife.62307 33289483 PMC7744096

[koaf126-B54] Pizer LI . The pathway and control of serine biosynthesis in *Escherichia coli*. J Biol Chem. 1963:238(12):3934–3944. 10.1016/S0021-9258(18)51809-3 14086727

[koaf126-B55] Possemato R, Marks KM, Shaul YD, Pacold ME, Kim D, Birsoy K, Sethumadhavan S, Woo H-K, Jang HG, Jha AK, et al Functional genomics reveal that the serine synthesis pathway is essential in breast cancer. Nature. 2011:476(7360):346–350. 10.1038/nature10350 21760589 PMC3353325

[koaf126-B56] Reid MA, Allen AE, Liu S, Liberti MV, Liu P, Liu X, Dai Z, Gao X, Wang Q, Liu Y, et al Serine synthesis through PHGDH coordinates nucleotide levels by maintaining central carbon metabolism. Nat Commun. 2018:9(1):5442. 10.1038/s41467-018-07868-6 30575741 PMC6303315

[koaf126-B57] Ren R, Jiang X, Zheng G, Zhao Y, Li J, Zhang X, Zhao X. ZmADT2 regulates maize kernel development via the auxin signaling pathway. Crop J. 2025:13(1):181–191. 10.1016/j.cj.2024.12.002

[koaf126-B58] Ros R, Muñoz-Bertomeu J, Krueger S. Serine in plants: biosynthesis, metabolism, and functions. Trends Plant Sci. 2014:19(9):564–569. 10.1016/j.tplants.2014.06.003 24999240

[koaf126-B59] Rosa-Téllez S, Alcántara-Enguídanos A, Martínez-Seidel F, Casatejada-Anchel R, Saeheng S, Bailes CL, Erban A, Barbosa-Medeiros D, Alepúz P, Matus JT, et al The serine-glycine-one-carbon metabolic network orchestrates changes in nitrogen and sulfur metabolism and shapes plant development. Plant Cell. 2024:36(2):404–426. 10.1093/plcell/koad256 37804096 PMC10827325

[koaf126-B60] Sage RF, Sage TL, Kocacinar F. Photorespiration and the evolution of C4 photosynthesis. Annu Rev Plant Biol. 2012:63(1):19–47. 10.1146/annurev-arplant-042811-105511 22404472

[koaf126-B61] Shu XE, Swanda RV, Qian S-B. Nutrient control of mRNA translation. Annu Rev Nutr. 2020:40(1):51–75. 10.1146/annurev-nutr-120919-041411 32631146

[koaf126-B62] Snell K . Enzymes of serine metabolism in normal, developing and neoplastic rat tissues. Adv Enzyme Regul. 1984:22:325–400. 10.1016/0065-2571(84)90021-9 6089514

[koaf126-B63] Snell K, Natsumeda Y, Eble JN, Glover JL, Weber G. Enzymic imbalance in serine metabolism in human colon carcinoma and rat sarcoma. Br J Cancer. 1988:57(1):87–90. 10.1038/bjc.1988.15 3126791 PMC2246686

[koaf126-B64] Snell K, Weber G. Enzymic imbalance in serine metabolism in rat hepatomas. Biochem J. 1986:233(2):617–620. 10.1042/bj2330617 3082329 PMC1153072

[koaf126-B65] Sun L, Song L, Wan Q, Wu G, Li X, Wang Y, Wang J, Liu Z, Zhong X, He X, et al cMyc-mediated activation of serine biosynthesis pathway is critical for cancer progression under nutrient deprivation conditions. Cell Res. 2015:25(4):429–444. 10.1038/cr.2015.33 25793315 PMC4387561

[koaf126-B66] Svenningsen SL, Kongstad M, Stenum TS, Muñoz-Gómez AJ, Sørensen MA. Transfer RNA is highly unstable during early amino acid starvation in *Escherichia coli*. Nucleic Acids Res. 2017:45(2):793–804. 10.1093/nar/gkw1169 27903898 PMC5314770

[koaf126-B67] Tajan M, Hennequart M, Cheung EC, Zani F, Hock AK, Legrave N, Maddocks ODK, Ridgway RA, Athineos D, Suárez-Bonnet A, et al Serine synthesis pathway inhibition cooperates with dietary serine and glycine limitation for cancer therapy. Nat Commun. 2021:12(1):366. 10.1038/s41467-020-20223-y 33446657 PMC7809039

[koaf126-B68] Thompson DM, Lu C, Green PJ, Parker R. tRNA cleavage is a conserved response to oxidative stress in eukaryotes. RNA. 2008:14(10):2095–2103. 10.1261/rna.1232808 18719243 PMC2553748

[koaf126-B69] Toujani W, Muñoz-Bertomeu J, Flores-Tornero M, Rosa-Téllez S, Anoman AD, Ros R. Identification of the phosphoglycerate dehydrogenase isoform EDA9 as the essential gene for embryo and male gametophyte development in *Arabidopsis*. Plant Signal Behav. 2013:8(11):e27207. 10.4161/psb.27207 24304635 PMC4091315

[koaf126-B70] Wang L, Czedik-Eysenberg A, Mertz RA, Si Y, Tohge T, Nunes-Nesi A, Arrivault S, Dedow LK, Bryant DW, Zhou W, et al Comparative analyses of C4 and C3 photosynthesis in developing leaves of maize and rice. Nat Biotechnol. 2014:32(11):1158–1165. 10.1038/nbt.3019 25306245

[koaf126-B71] Wang Q, Wang M, Chen J, Qi W, Lai J, Ma Z, Song R. ENB1 encodes a cellulose synthase 5 that directs synthesis of cell wall ingrowths in maize basal endosperm transfer cells. Plant Cell. 2022:34(3):1054–1074. 10.1093/plcell/koab312 34935984 PMC8894971

[koaf126-B72] Xiong Y, McCormack M, Li L, Hall Q, Xiang C, Sheen J. Glucose–TOR signalling reprograms the transcriptome and activates meristems. Nature. 2013:496(7444):181–186. 10.1038/nature12030 23542588 PMC4140196

[koaf126-B73] Xiong Y, Sheen J. Rapamycin and glucose-Target of Rapamycin (TOR) protein signaling in plants. J Biol Chem. 2012:287(4):2836–2842. 10.1074/jbc.M111.300749 22134914 PMC3268441

[koaf126-B74] Yang M, Vousden KH. Serine and one-carbon metabolism in cancer. Nat Rev Cancer. 2016:16(10):650–662. 10.1038/nrc.2016.81 27634448

[koaf126-B75] Yi F, Gu W, Chen J, Song N, Gao X, Zhang X, Zhou Y, Ma X, Song W, Zhao H, et al High temporal-resolution transcriptome landscape of early maize seed development. Plant Cell. 2019:31(5):974–992. 10.1105/tpc.18.00961 30914497 PMC6533015

[koaf126-B76] Yuan W, Guo S, Gao J, Zhong M, Yan G, Wu W, Chao Y, Jiang Y. General control nonderepressible 2 (GCN2) kinase inhibits Target of Rapamycin Complex 1 in response to amino acid starvation in *Saccharomyces cerevisiae*. J Biol Chem. 2017:292(7):2660–2669. 10.1074/jbc.M116.772194 28057755 PMC5314164

[koaf126-B77] Zhan J, Thakare D, Ma C, Lloyd A, Nixon NM, Arakaki AM, Burnett WJ, Logan KO, Wang D, Wang X, et al RNA sequencing of laser-capture microdissected compartments of the maize kernel identifies regulatory modules associated with endosperm cell differentiation. Plant Cell. 2015:27(3):513–531. 10.1105/tpc.114.135657 25783031 PMC4558669

[koaf126-B78] Zhang Y, Wang Y, Kanyuka K, Parry MA, Powers SJ, Halford NG. GCN2-dependent phosphorylation of eukaryotic translation initiation factor-2alpha in *Arabidopsis*. J Exp Bot. 2008:59(11):3131–3141. 10.1093/jxb/ern169 18603615 PMC2504353

[koaf126-B79] Zhang Z, Dong J, Ji C, Wu Y, Messing J. NAC-type transcription factors regulate accumulation of starch and protein in maize seeds. Proc Natl Acad Sci U S A. 2019:116(23):11223–11228. 10.1073/pnas.1904995116 31110006 PMC6561305

[koaf126-B80] Zhigailov AV, Alexandrova AM, Nizkorodova AS, Stanbekova GE, Kryldakov RV, Karpova OV, Polimbetova NS, Halford NG, Iskakov BK. Evidence that phosphorylation of the α-subunit of eIF2 does not essentially inhibit mRNA translation in wheat germ cell-free system. Front Plant Sci. 2020:11:936. 10.3389/fpls.2020.00936 32655610 PMC7324750

[koaf126-B81] Zhigailov AV, Stanbekova GE, Nizkorodova AS, Galiakparov NN, Gritsenko DA, Polimbetova NS, Halford NG, Iskakov BK. Phosphorylation of the alpha-subunit of plant eukaryotic initiation factor 2 prevents its association with polysomes but does not considerably suppress protein synthesis. Plant Sci. 2022:317:111190. 10.1016/j.plantsci.2022.111190 35193739

[koaf126-B82] Zimmermann SE, Benstein RM, Flores-Tornero M, Blau S, Anoman AD, Rosa-Téllez S, Gerlich SC, Salem MA, Alseekh S, Kopriva S, et al The phosphorylated pathway of serine biosynthesis links plant growth with nitrogen metabolism. Plant Physiol. 2021:186(3):1487–1506. 10.1093/plphys/kiab167 34624108 PMC8260141

