# Supplementary material for: Phosphoglycerate dehydrogenase is required for kernel development and defines a predominant serine synthesis pathway in maize
Source: Plant Cell. 2025 May 22;37(6):koaf126. doi: 10.1093/plcell/koaf126 (PMC12164754; doi:10.1093/plcell/koaf126)
Supplement: koaf126_Supplementary_Data [file koaf126_supplementary_data.zip › Supplementary Figures and Figure legends.pdf]

	Ear1	Ear2	Ear3
WT	154	195	236
dek20	56	68	83
χ^2	0.505	0.52	0.18

Supplementary Figure S1. Chi-square Tests of the Defective Kernel Phenotype in *dek20*/+ Selfed Ears (Support Figure 1)**.** $\chi^2 < 3.84$, WT and *dek20* kernels at a ratio of 3 : 1.

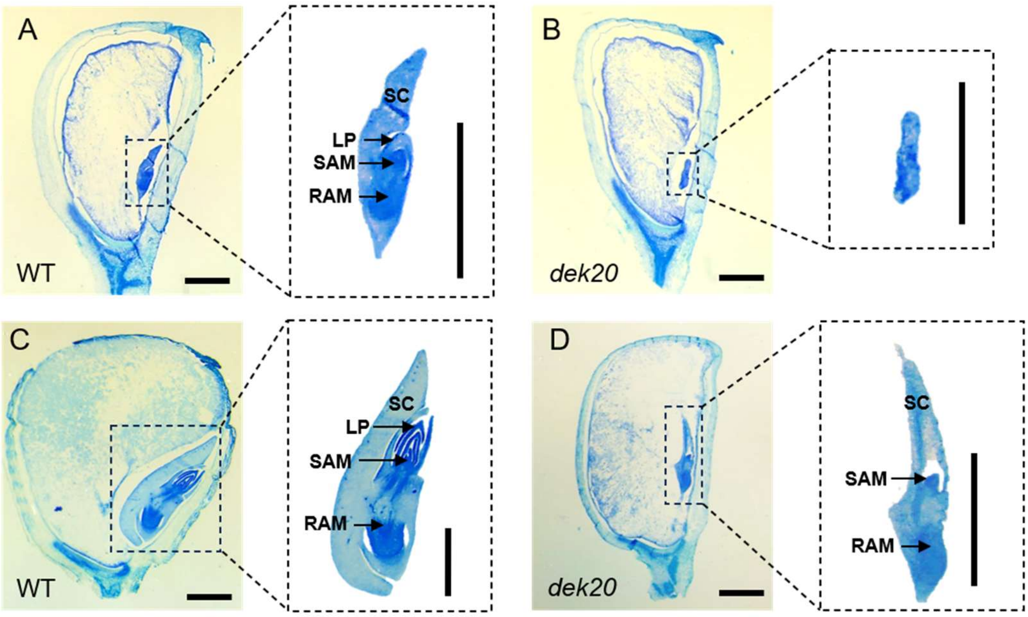

Supplementary Figure S2. Histological Analysis of Developing WT and *dek20* Kernels (Support Figure 1)**.**

(A) and (B) Longitudinal Paraffin Sections of WT and *dek20* Kernels at 10 DAP. Embryos are enlarged to show the details (Images were digitally extracted for comparison). SC, scutellum; LP, leaf primordia; SAM, shoot apical meristem; RAM, root apical meristem. Scale bar = 0.5 mm.

(C) and (D) Longitudinal Paraffin Sections of WT and *dek20* Kernels at 18 DAP. Embryos are enlarged to show the details (Images were digitally extracted for comparison). Scale bar = 0.5 mm.

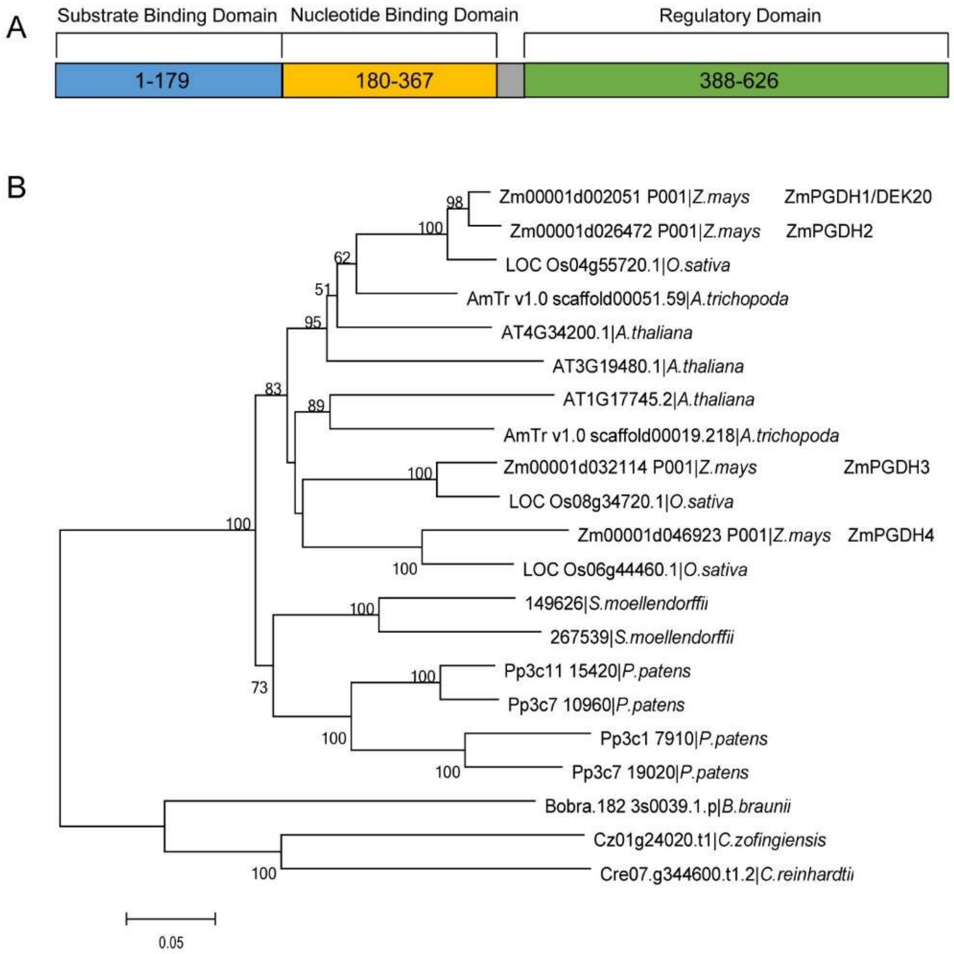

Supplementary Figure S3. Phylogenetic Analysis of *Dek20* (Support Figure 3)**.**

(A) Domain analysis of DEK20 protein.

(B) Phylogenetic relationships of maize DEK20 and its homologs in other species. Multiple sequence alignment was performed using the MUSCLE algorithm in the MEGA 6.0 software package. The phylogenetic tree was constructed using the neighbor-joining method with 1000 bootstrap replicates to assess node support. Scale bar = average number of amino acid substitutions per site.

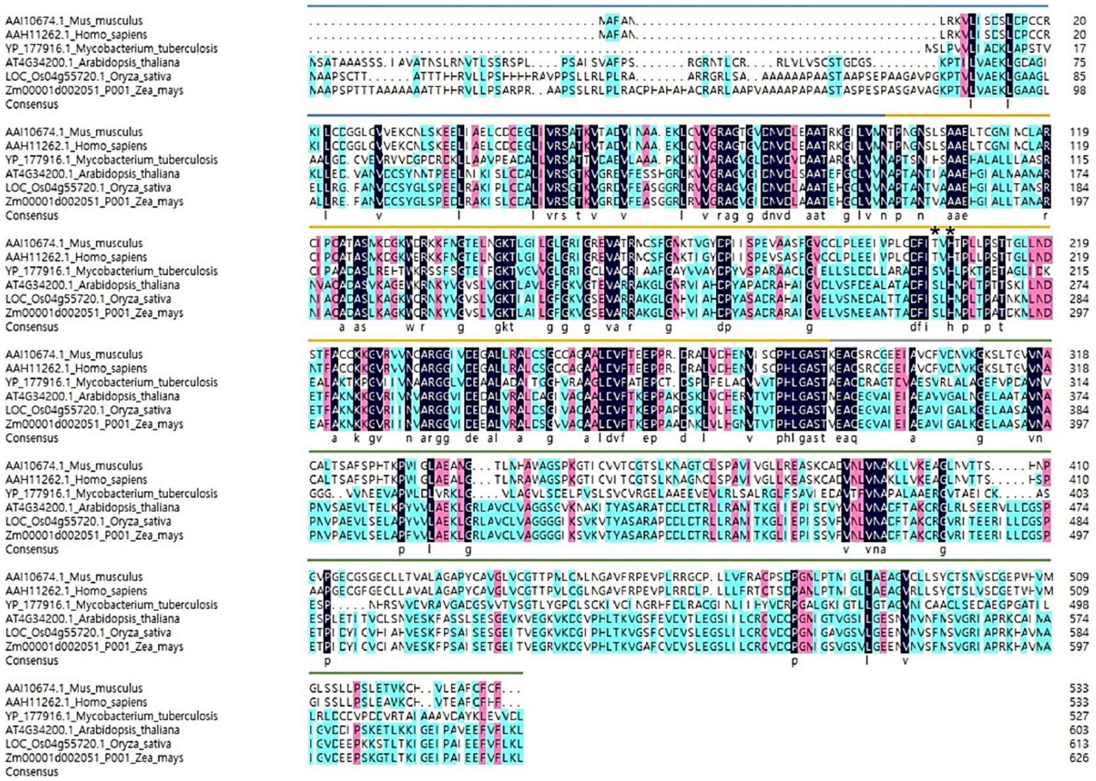

Supplementary Figure S4. Amino Acid Sequence Alignment of DEK20 and its Homologs from Representative Species (Support Figure 3)**.**

Protein domains are highlighted with colored lines. Substrate binding domain (SBD, residues 1-179), blue; nucleotide binding domain (NBD, residues 180-367), yellow; regulatory domain (RBD, residues 388-626), green. Conserved residues are highlighted in dark blue. The asterisks indicate the 282nd residue Ser and 284th residue His of DEK20.

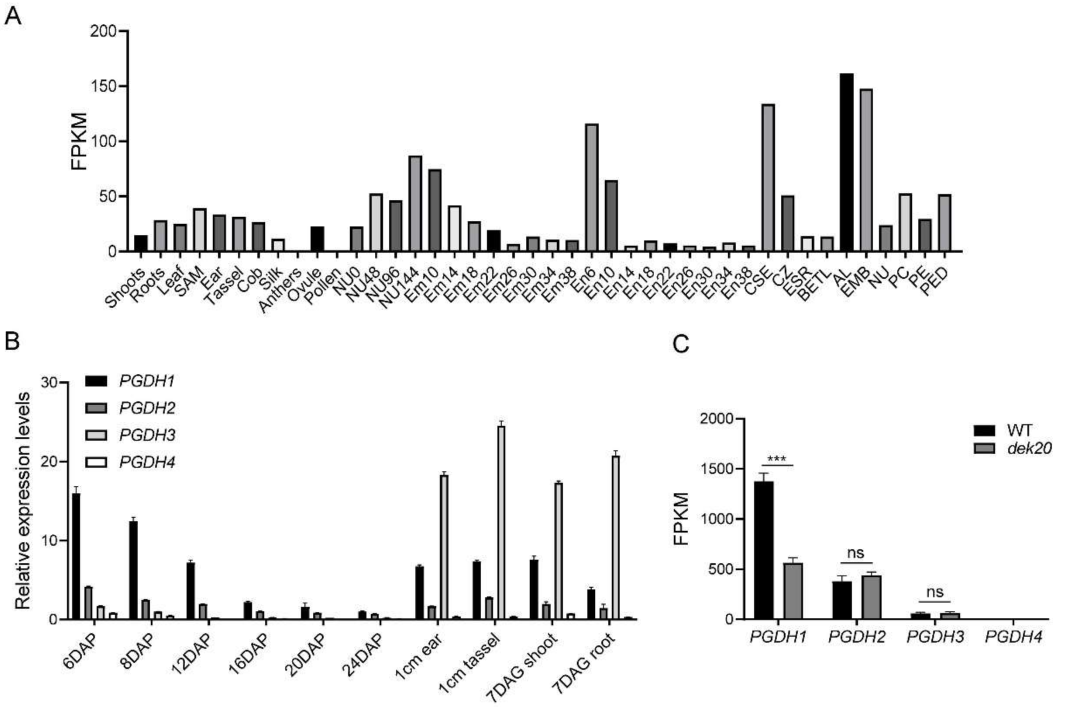

Supplementary Figure S5. Expression Pattern of *Dek20* Homologous Genes (Support Figure 3A).

(A) The temporal and spatial expression analysis of *Dek20* from public transcriptome data (Pubmed-25037214).

(B) The transcriptional expression analysis of maize *Dek20* homologous genes by RT-qPCR. Three independent RNA samples from different plants were used as biological replicates. DAP means days after pollination. The whole kernel with embryo and endosperm is used. DAG means days after gemination. Values are means ± SD (n = 3).

(C) The transcriptional expression analysis of maize *Dek20* homologous genes in *dek20*. Values are means ± SD (n = 3, kernels from three independent ears, ***p < 0.001, ns, no significant difference as determined by two-tailed t test).

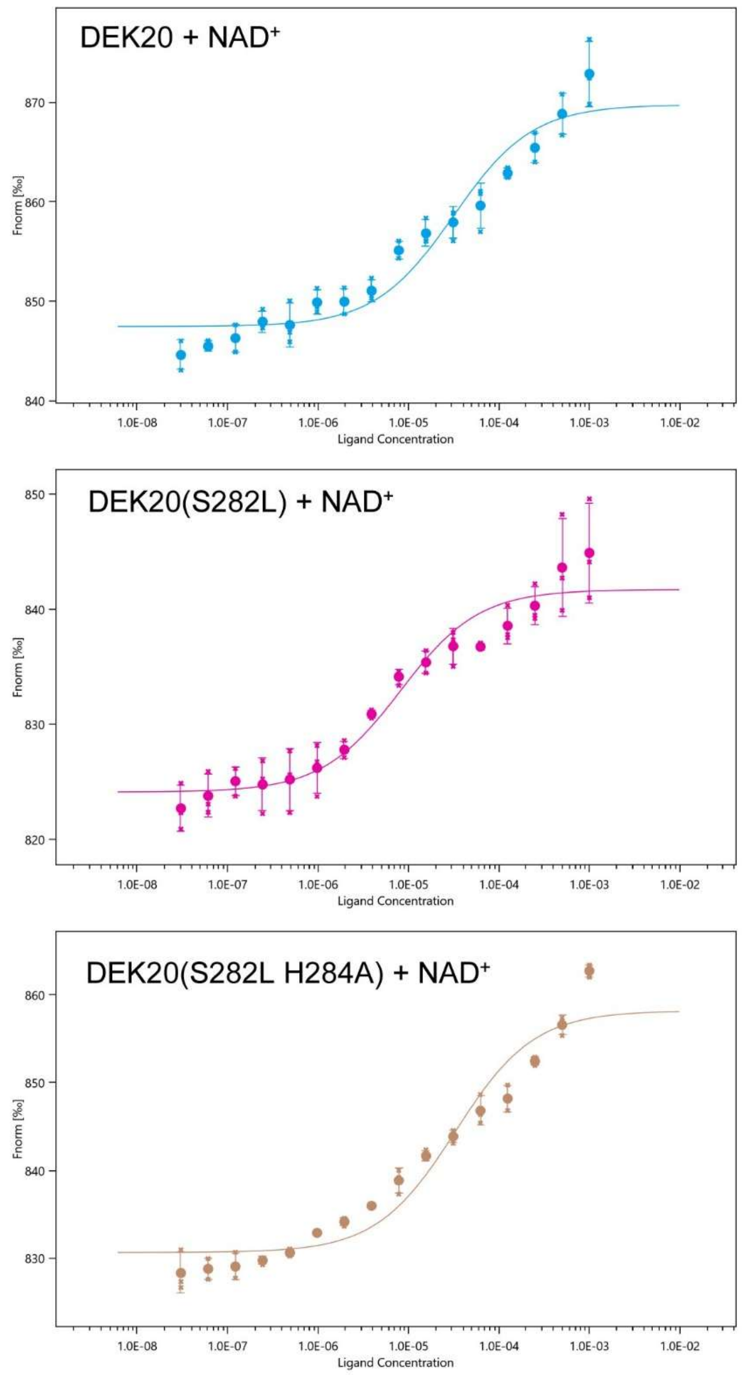

Supplementary Figure S6. Microscale Thermophoresis Analysis of the Affinity to NAD$^+$ for DEK20, DEK20 (S282L) and DEK20(S282L H284A) Proteins (Support Figure 5F)**.**

Fourteen dilutions of NAD$^+$ were used, and the curves were fitted using the data of three independent experiments. Values are means ± SD (*n* = 3 biologically independent experiments).

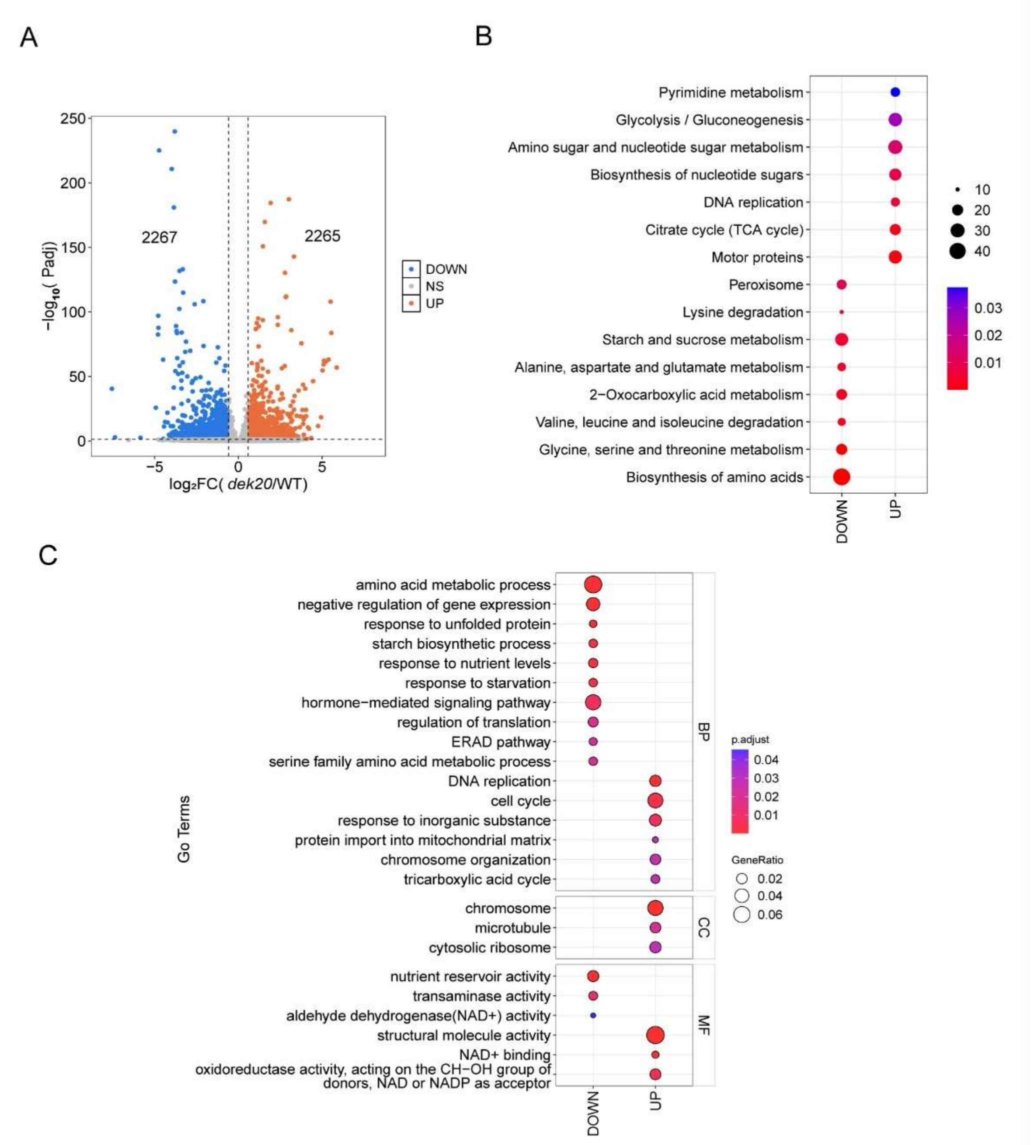

Supplementary Figure S7. Transcriptome Analysis of *dek20* (Support Figure 9)**.**

(A) Volcano plot of differentially expressed genes (DEGs) in *dek20* versus WT kernels. Differential expression defined by |Fold change|≥1.5 and padj < 0.05.

(B) KEGG enrichment analysis of DEGs in *dek20* versus WT kernels. Color scale indicates *p* value thresholds; dot size indicates gene number for each pathway.

(C) GO enrichment analysis of DEGs in *dek20* versus WT kernels. Color scale indicates *p* value thresholds; dot size indicates gene ratio for each GO term, which helps assess the relative significance of each GO term, with higher values indicating a stronger correlation of that term in the enrichment analysis.

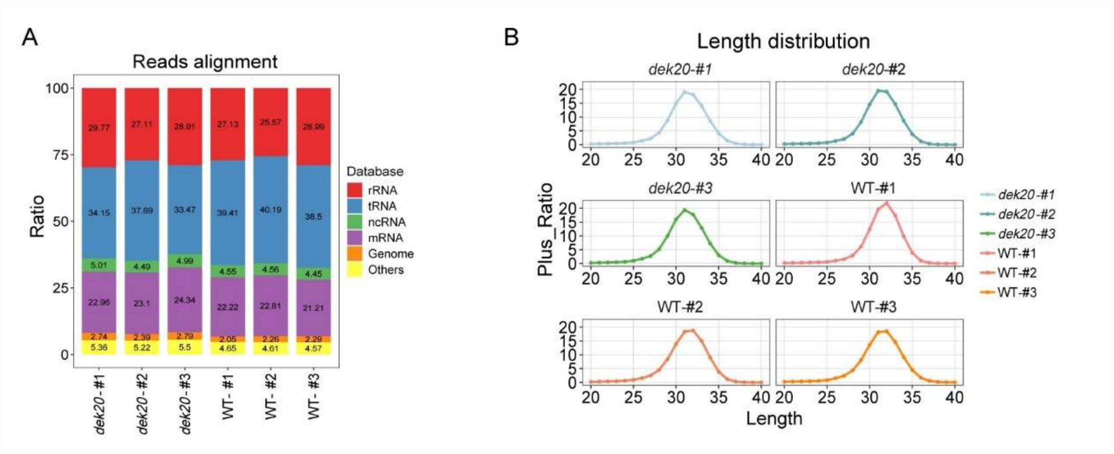

Supplementary Figure S8. Ribosome Profiling Sequencing Statistics of WT and *dek20* Kernels (Support Figures 7 and 9)**.**

(A) The distribution of ribosome protected fragments (RPFs) from different origins.

(B) Length distribution of RPFs from 3 independent replicates (kernels from three independent ears).

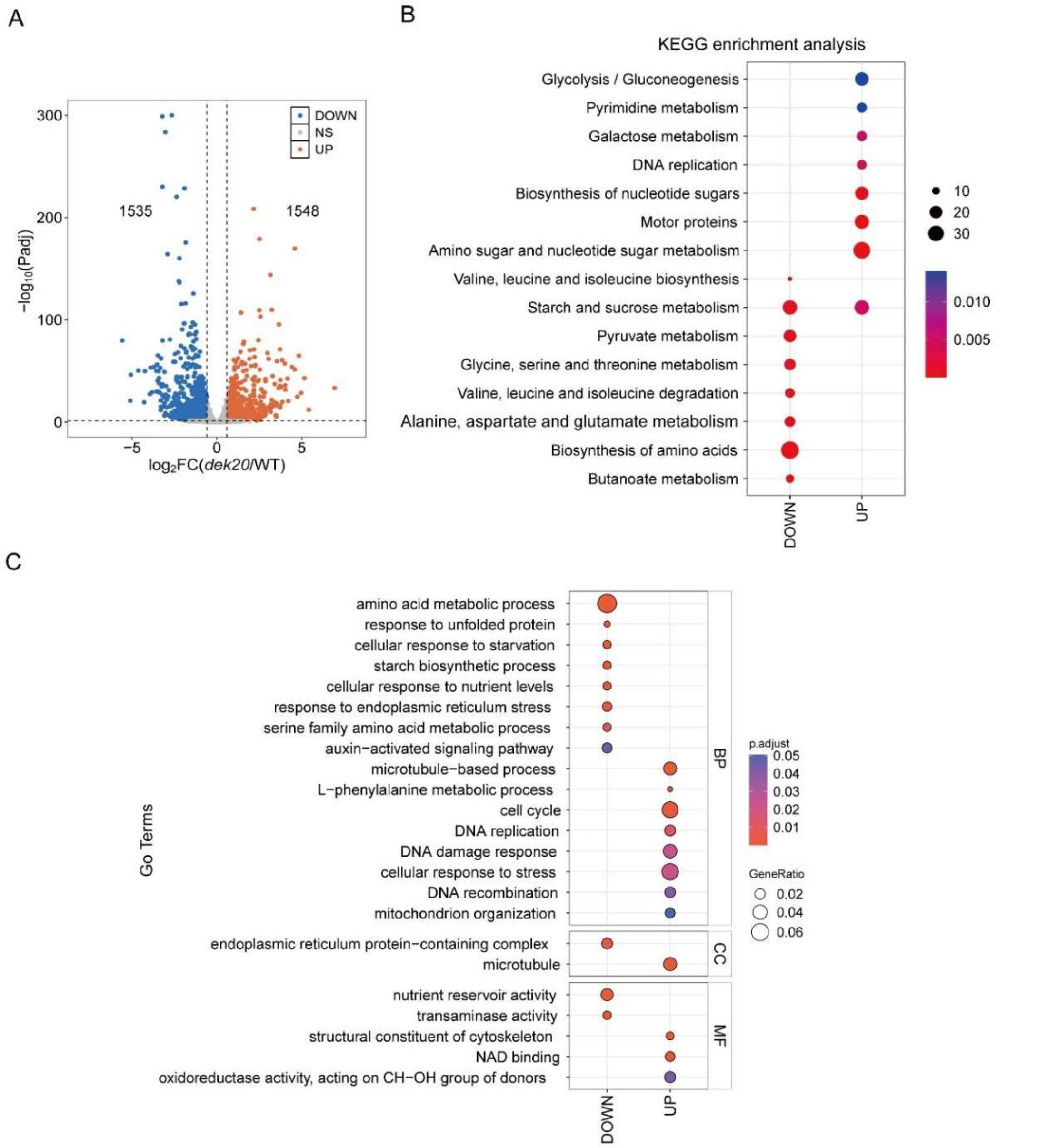

Supplementary Figure S9. Translatome Analysis of *dek20* (Support Figures 7 and 9)**.**

(A) Volcano plot of differentially translated genes (DTGs) in *dek20* versus WT kernels. Differential expression defined by |Fold change|≥1.5 and padj < 0.05.

(B) KEGG enrichment analysis of DTGs in *dek20* versus WT kernels. Color scale indicates p value thresholds; dot size indicates gene number for each pathway.

(C) GO enrichment analysis of DTGs in *dek20* versus WT kernels. Color scale indicates *p* value thresholds; dot size indicates gene ratio for each GO term, which helps assess the relative significance of each GO term, with higher values indicating a stronger correlation of that term in the enrichment analysis.

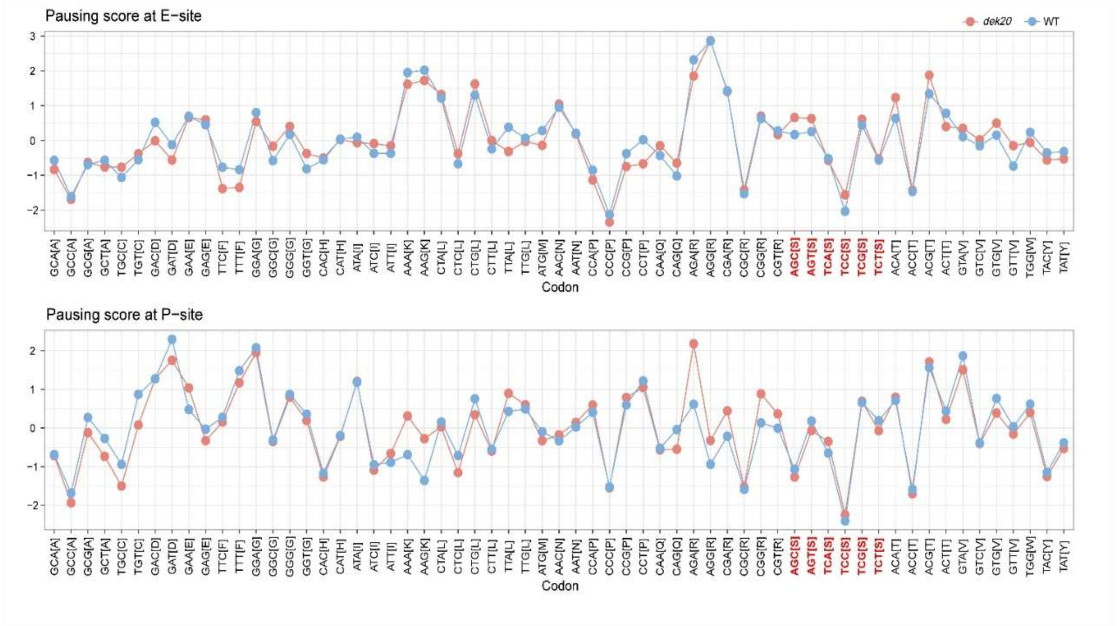

Supplementary Figure S10. Plots of Pausing Scores for Individual Codon at the Ribosomal E and P Sites in *dek20* versus WT Kernels (Support Figure 7D). Plots of pausing scores for individual codon at the ribosomal E (upper panel) and P (lower panel) sites. Codons for serine residue are label in red. Average scores of 3 independent replicates (kernels from three independent ears).

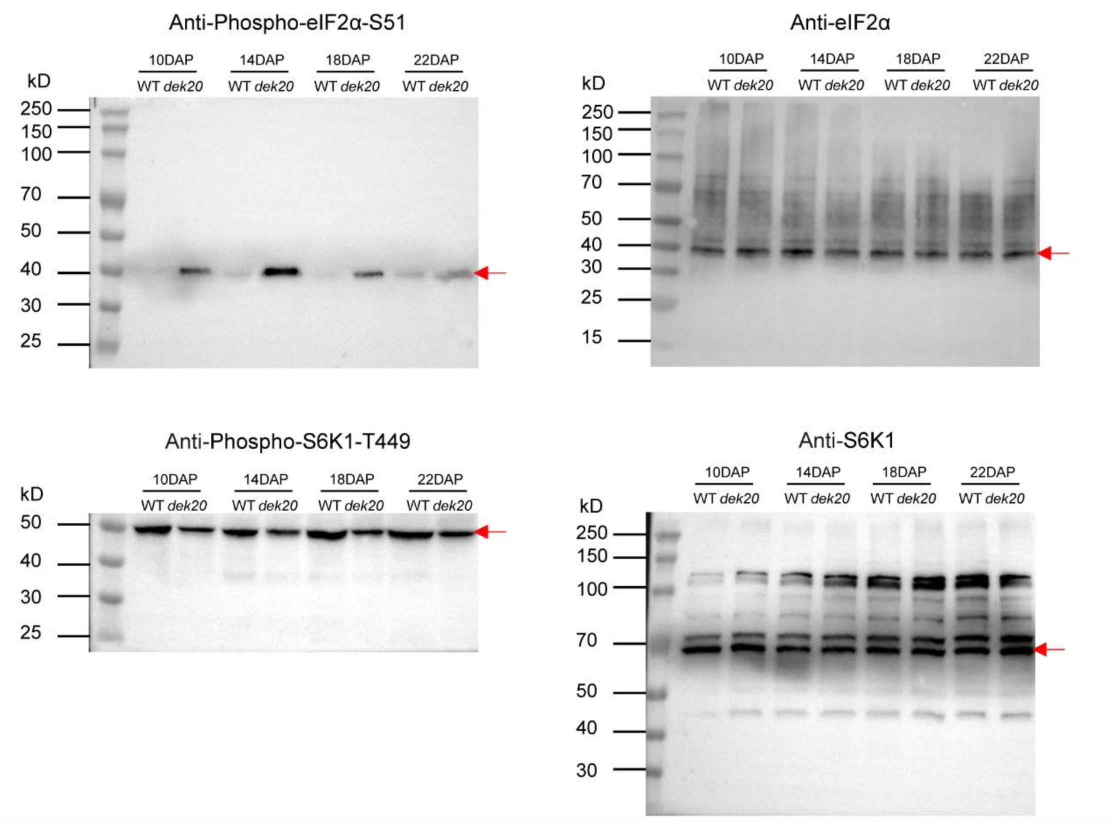

Supplementary Figure S11. eIF2α and S6K1 Phosphorylation Analysis. These blots are un-cropped versions of those in Figure 10A. Migration of the endogenous unphosphorylated S6K is unusual, which is also found and verified in a published literature (Pubmed-34765910). Red arrows indicate target protein.

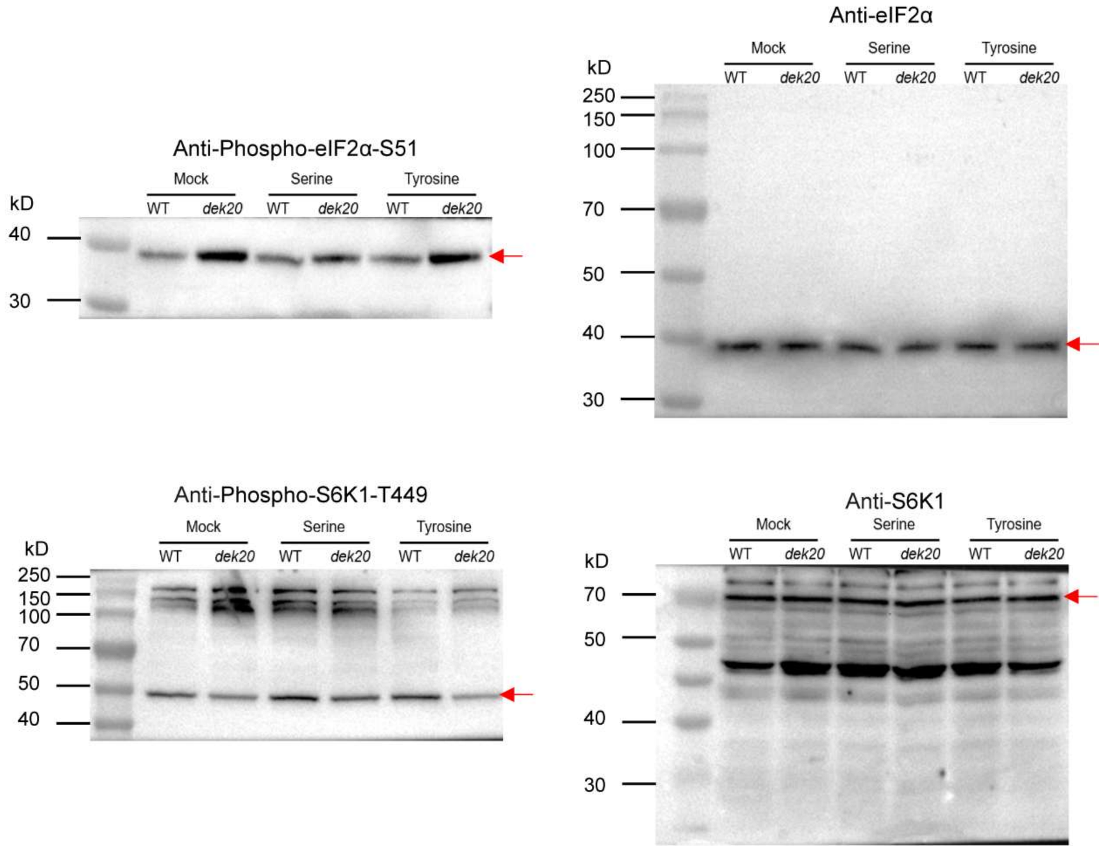

Supplementary Figure S12. eIF2α and S6K1 Phosphorylation Analysis after Cultivated in Medium Supplemented with 3 mM L-Serine or 3 mM L-Tyrosine. These blots are un-cropped versions of those in Figure 10B. Migration of the endogenous unphosphorylated S6K is unusual, which is also found and verified in a published literature (Pubmed-34765910). Red arrows indicate target protein. n = 30, biologically independent kernels.

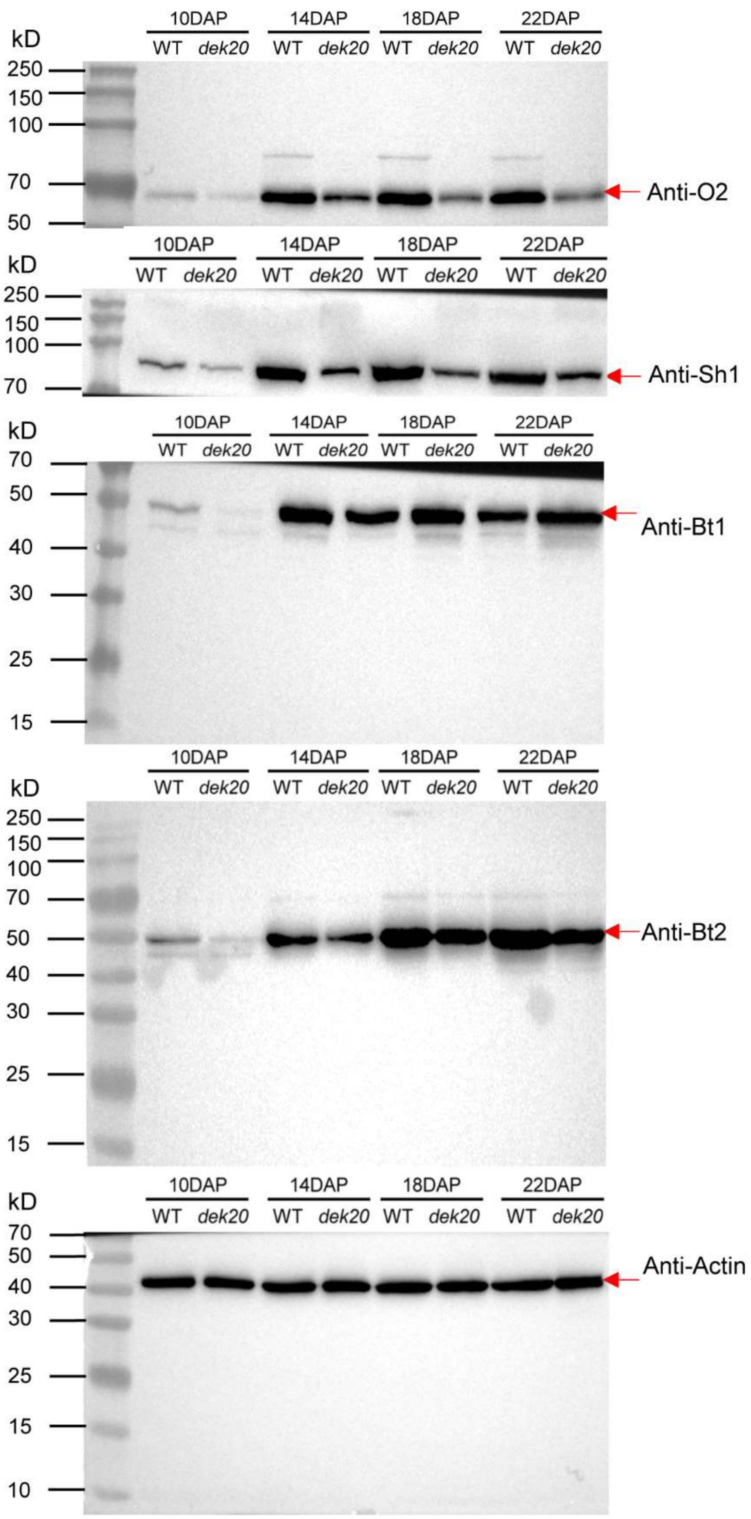

Supplementary Figure S13. Immunoblotting Analysis of Proteins for Starch and Protein Synthesis in *dek20*. These blots are un-cropped versions of those in Figure 11A. Red arrows indicate target protein.

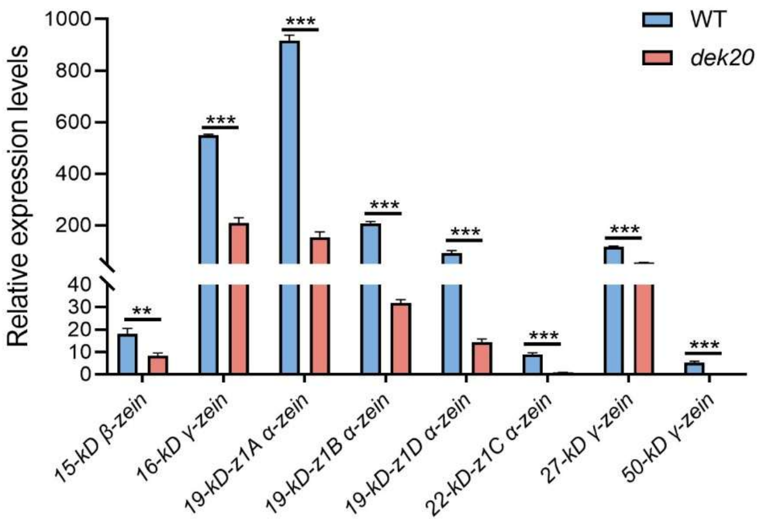

Supplementary Figure S14. Expression Analysis of *Zein* Genes (Support Figure 11A)**.** Values are means ±SD (*n* = 3, kernels from three independent ears, ***p* < 0.01; ****p* < 0.001 as determined by two-tailed *t* test)..

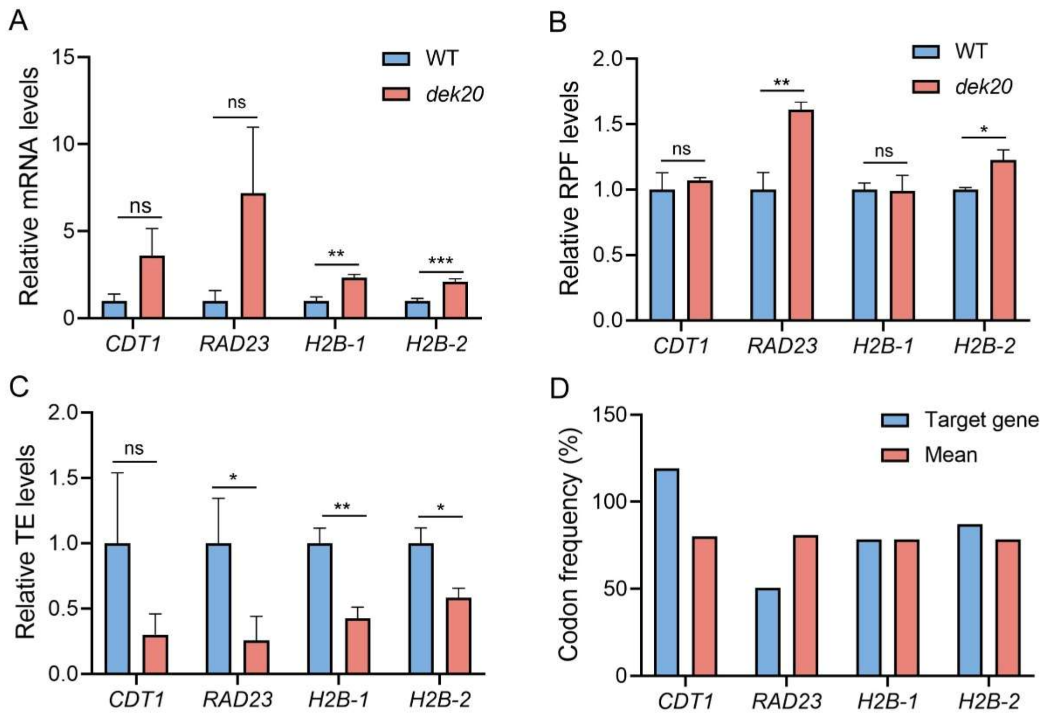

Supplementary Figure S15. Translation Analysis of Cell Cycle Related Genes (Support Figure 12)**.**

(A) RNA-seq expression analysis of *CDT1*, *RAD23*, *H2B-1*, and *H2B-2* in *dek20*. Values are means ± SD (n = 3, kernels from three independent ears; **$p < 0.01$, ***$p < 0.001$; ns, no significant difference as determined by two-tailed t test).

(B) Ribo-seq analysis of *CDT1*, *RAD23*, *H2B-1*, and *H2B-2* in *dek20*. Values are means ± SD (n = 3, kernels from three independent ears; *$p < 0.05$, **$p < 0.01$; ns, no significant difference as determined by two-tailed t test).

(C) Translation efficiency analysis of *CDT1*, *RAD23*, *H2B-1*, and *H2B-2* in *dek20*. Values are means ± SD (n = 3, kernels from three independent ears; *$p < 0.05$, **$p < 0.01$; ns, no significant difference as determined by two-tailed t test).

(D) Analysis of Ser codons in CDT1, RAD23, H2B-1, and H2B-2 proteins. Average scores of 3 replicates (kernels from three independent ears).
